# YAP/TAZ: Molecular pathway and disease therapy

**DOI:** 10.1002/mco2.340

**Published:** 2023-08-09

**Authors:** Yuzi Wei, Victoria Lee Zhi Hui, Yilin Chen, Ruiying Han, Xianglong Han, Yongwen Guo

**Affiliations:** ^1^ State Key Laboratory of Oral Diseases & National Clinical Research Center for Oral Diseases, West China Hospital of Stomatology Sichuan University Chengdu Sichuan China; ^2^ Department of Orthodontics West China Hospital of Stomatology Sichuan University Chengdu Sichuan China; ^3^ Department of Orthodontics Lanzhou Stomatological Hospital Lanzhou Gansu China

**Keywords:** atherosclerosis, cancer, fibrosis, metabolism, regeneration, YAP/TAZ

## Abstract

The Yes‐associated protein and its transcriptional coactivator with PDZ‐binding motif (YAP/TAZ) are two homologous transcriptional coactivators that lie at the center of a key regulatory network of Hippo, Wnt, GPCR, estrogen, mechanical, and metabolism signaling. YAP/TAZ influences the expressions of downstream genes and proteins as well as enzyme activity in metabolic cycles, cell proliferation, inflammatory factor expression, and the transdifferentiation of fibroblasts into myofibroblasts. YAP/TAZ can also be regulated through epigenetic regulation and posttranslational modifications. Consequently, the regulatory function of these mechanisms implicates YAP/TAZ in the pathogenesis of metabolism‐related diseases, atherosclerosis, fibrosis, and the delicate equilibrium between cancer progression and organ regeneration. As such, there arises a pressing need for thorough investigation of YAP/TAZ in clinical settings. In this paper, we aim to elucidate the signaling pathways that regulate YAP/TAZ and explore the mechanisms of YAP/TAZ‐induce diseases and their potential therapeutic interventions. Furthermore, we summarize the current clinical studies investigating treatments targeting YAP/TAZ. We also address the limitations of existing research on YAP/TAZ and propose future directions for research. In conclusion, this review aims to provide fresh insights into the signaling mediated by YAP/TAZ and identify potential therapeutic targets to present innovative solutions to overcome the challenges associated with YAP/TAZ.

## Graphical abstract

YAP/TAZ lies at the center of regulatory networks as a core component. At present, YAP/TAZ is considered to be regulated by Hippo signaling, Wnt signaling, GPCR signaling, estrogen signaling, mechanical signaling, and metabolism signaling. In the functions of various signaling pathways, YAP/TAZ participates in the regeneration medicine as well as the development of diverse diseases including metabolism‐related diseases, cancers, organ fibrosis. Therefore, YAP/TAZ can serve as a potential treatment target in multiple organ diseases.

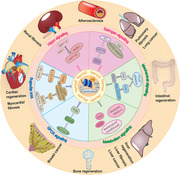

## 1 INTRODUCTION

**FIGURE 1:**
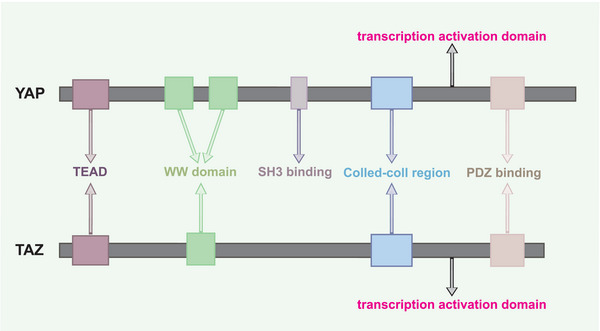
The structure of YAP/TAZ. At the N‐terminal, YAP/TAZ all have a TEAD binding region and all have a transcription activation domain at the C‐terminal, including PDZ binding and Coiled‐coil region. Comparing with the TAZ, the YAP also has a SH3 binding region and YAP2 has one more WW domain.

Yes‐associated protein (YAP) and transcriptional coactivator with PDZ‐binding motif (TAZ) are transcriptional regulators that were initially discovered in Drosophila in 1995. YAP has two major isoforms: YAP1, which contains one WW domain, and YAP2, which contains two WW domains.^1^, ^2^ TAZ, a 43 kDa protein, is different from YAP2 in that it lacks the SH3‐BM proline‐rich region found in the 65 kDa YAP protein and only has one WW domain presenting.^3^ This paper focuses on YAP1. The common structural elements found in YAP/TAZ include the WW domain, C‐terminal transcriptional activation domain, and TEA domain‐containing sequence‐specific transcription factors (TEAD) binding region at the N‐terminus (Figure 1). In terms of mechanism, activated YAP/TAZ translocates from the cytoplasm to the nucleus, where it binds to TEAD in the chromosome, thereby regulating the expression of target genes and exerting diverse effects. Thus, YAP/TAZ is closely associated with enzyme activity, cell proliferation, differentiation, tissue, and organ growth and can mediate the production of proinflammatory factors.^3^

In recent years, an increasing number of studies have discovered that YAP/TAZ can be regulated by various mechanisms, ranging from diverse signaling pathways to epigenetic regulation and protein modification that influences the biological functions of cells, tissues, and organs. Consequently, these proteins play a role in different diseases and organ regeneration. Alongside advancing research on YAP/TAZ, there is also a growing exploration of drugs that target them. This review aims to provide a comprehensive summary of how complex signaling pathway networks regulate YAP/TAZ and how epigenetic regulation and posttranslational modifications impact their activity. Furthermore, this article delves into the mechanisms through which YAP/TAZ contributes to metabolic diseases, cancer, atherosclerosis and organ fibrosis. It also discusses the potential for YAP/TAZ‐induced organ regeneration and presents the latest progress in targeted YAP/TAZ drug therapy. Ultimately, these insights and strategies aim to advance the treatment of YAP/TAZ‐mediated diseases.

## 2 THE REGULATION OF YAP/TAZ

**FIGURE 2:**
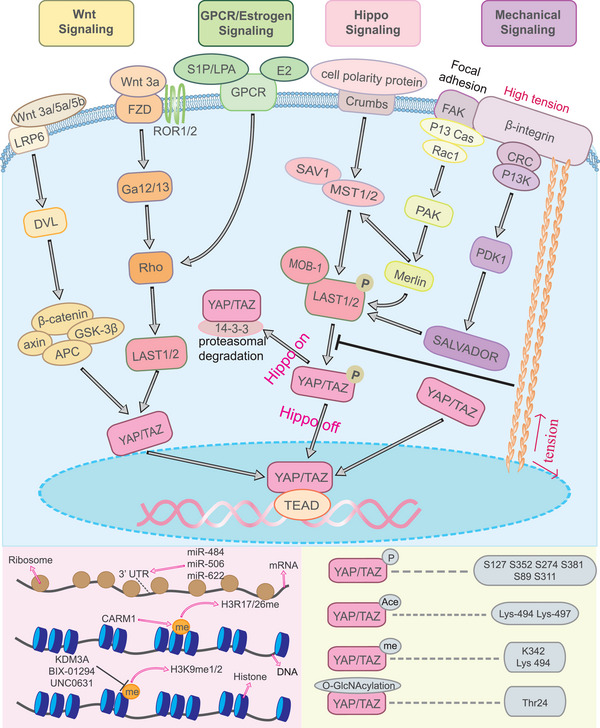
The main signaling pathways of YAP/TAZ. YAP/TAZ can be regulated by Hippo signaling, Wnt signaling, GPCR signaling, estrogen signaling, and mechanical signaling. In the Hippo signaling pathway, the phosphorylated MST1/2 interacts with SAV1 to phosphorylate and activate the LATS1/2, then the activated LAST1/2 phosphorylates and inhibits YAP/TAZ. Wnt signaling has two pathways to regulate the YAP/TAZ. One way is to activate YAP/TAZ through LATS1/2 inhibited by Rho while the other is to modulate YAP/TAZ by β‐catenin complex. And the regulations of GPCR signaling and Estrogen signaling are almost similar as the previous one of Wnt signaling. YAP/TAZ can serve as mechanotransduction to participant in mechanical signaling. Under the stimuli of FA, YAP/TAZ is regulated through two Hippo‐dependent pathways: β1‐integrin–FAK–SRC–PI3K–PDK1 signaling pathway and the β1‐integrin–FAK–P130–Cas–Rac1–PAK–Merlin pathway. In the Hippo‐independent pathway, mechanical signaling is transmitted to the nucleus mediated by stress fiber. The unphosphorylated and uninhibited YAP/TAZ is able to proceed the nuclear translocation.

In fact, YAP/TAZ occupies a central position within a complex network of signaling pathways, with significant crosstalk occurring between each pathway (Figure 2). Once activated, YAP/TAZ translocates into the nucleus and binds to TEAD to mediate the expression of downstream target genes. Besides the involvement of various signaling pathways, recent research has highlighted the regulatory impact of epigenetic modifications and posttranslational modifications on YAP/TAZ activity, which subsequently affects its function.

### 2.1 YAP/TAZ regulated by Hippo signaling

YAP/TAZ serves as a key downstream mediator in the Hippo signaling pathway. The core components of the classical Hippo signaling pathway include the MST1/2 complex (serine/threonine kinases 3/4), which is the ortholog of Drosophila Hippo, SAV1 (scaffold protein Salvador homolog 1), the LATS1/2 complex (large tumor suppressor kinases 1/2), corresponding to Drosophila Wts, the cofactor MOB1A/B (MOB kinase activator scaffolding proteins), and the YAP/TAZ.^4^ Through cooperation, MST1/2 and SAV1 phosphorylate and activate LATS1/2, leading to the formation of an active LATS1/2/MOB‐1 complex that phosphorylates and inhibits YAP/TAZ. The Phosphorylated YAP/TAZ binds to the 14‐3‐3 protein, triggering proteasomal degradation. Furthermore, casein kinase 1δ (CK1δ)/1ε can additionally phosphorylate the YAP/TAZ, while the SCF β‐TRCP E3 ubiquitin ligase ubiquitinates the complex, resulting in the degradation of YAP/TAZ by the target protein.^5^ Interestingly, mitogen‐activated protein kinases (MAPK4K) can also impact phosphorylation and activation of LATS1/LATS2 in the absence of hippo signaling.^6^ In contrast to phosphorylated YAP/TAZ, unphosphorylated YAP/TAZ accumulates in the nucleus and binds to TEAD, promoting the expression of downstream genes.

Upstream inputs affecting the Hippo signaling pathway include cell–cell junction/cell polarity‐related protein complexes, as well as physical controls of YAP/TAZ. Merlin, a crucial upstream regulator within the core Hippo kinase, acts as an efficient inducer in cell–cell junction/cell polarity‐related protein complexes, which helps to the inhibition of YAP/TAZ.^7^ Similarly, Scribble (SCRB), functioning as a scaffold, facilitates the assembly of the MST/LATS/TAZ complex, thereby activating the core kinases MST and LATS, leading to the dampening of YAP/TAZ activity.^8^ Furthermore, the tumor suppressor neurofibromin 2 (NF2) collaborates with kidney and brain protein, which possesses WW and C2 domains, forming a complex that activates LATS1/2.^9^ NF2 also interacts with angiomotin, situated on the plasma membrane, to facilitate the recruitment of LATS kinases. Intriguingly, phosphorylated LATS can reciprocally influence angiomotin, establishing a powerful positive feedback in the Hippo pathway.^10^ By facilitating the assembly of LATS1/2 at the cell plasma membrane, NF2 can also encourage the phosphorylation of LATS1/2 through MST1/2‐mediated mechanisms.^11^

Furthermore, recent studies have highlighted additional signals that regulate the Hippo signaling pathway through physical mechanisms. Utilizing tauroursodeoxycholic acid, an endoplasmic reticulum stress inducer, initially causes a decrease in YAP phosphorylation, followed by an increase.^12^ Oxygen deprivation has also been observed to impact the activity of YAP/TAZ. Under anoxic conditions, due to LATS1/2 latency, the phosphorylation and inhibition of YAP/TAZ could be attenuated, which suggests an increase in YAP/TAZ activity.^13^ Additionally, the SIAH2 ubiquitin E3 ligase has been identified as a significant regulator of the Hippo pathway during hypoxia conditions.^14^

The Hippo pathway encompasses a multitude of upstream signaling events and has significant implications for downstream responses. Proper regulation of the Hippo pathway is crucial for tissue/organ development and cell proliferation, as its dysregulation can result in tissue/organ enlargement, which can then contribute to tumorigenesis.^15^, ^16^

In conclusion, the Hippo signaling pathway operates within a complex network environment and interacts with other signal pathways. Its central role is intimately tied to the regular growth and development of the body, as well as the physiological functions of cells. In order to fully understand the Hippo pathway's complex roles and produce fresh insights for the prevention and treatment of particular illnesses, more study on this route is required.

### 2.2 YAP/TAZ regulated by Wnt signaling

Recently, it has been demonstrated that YAP/TAZ serves important roles in gene expression, cell migration, and other processes through the regulation of Wnt signaling pathway.^17^ As for the TAZ, the phosphorylated β‐catenin can promote its degradation, whereas Wnt signaling stabilizes TAZ without affecting the stability of the YAP.^18^ Classic Wnt signaling interacts with β‐catenin and Wnt can combine with lipoprotein receptors (LRP) and Frizzled (FZD) family receptors to inhibit the β‐catenin destruction complex. The β‐catenin destruction complex consists of adenomatous polyposis coli, Axin, casein kinase I (CK1), glycogen synthase kinase‐3β (GSK‐3β) and is able to phosphorylate β‐catenin, and then the phosphorylated β‐catenin is ubiquitylated and degraded. Activation of the Wnt signaling pathway facilitates the dephosphorylation of β‐catenin, allowing its translocation into the nucleus. Notably, a study discovered a new alternative Wnt–YAP/TAZ signaling axis, which consisted of Wnt–FZD/ROR–Gα12/13‐Rho–Lats1/2–YAP/TAZ–TEAD.^19^ So, either Wnt signaling or its ligand plays a vital role in regulating YAP/TAZ activity.

Wnt5a/5b has a significant impact on the phosphorylation of Dishaveled (DVL), which can activate the alternative Wnt/YAP/TAZ signaling axis.^20^ Interestingly, YAP/TAZ makes an impact on inhibiting the DVL and further inhibiting the Wnt signaling, creating a negative feedback loop.^21^ Additionally, Wnt5a/5b acts as a downstream effector of YAP/TAZ, establishing a positive feedback loop.^22^ Furthermore, Wnt3a has the ability to activate YAP/TAZ, which in turn modulates caudal type homeobox 2（CDX2） expression via the Wnt/YAP/TAZ signaling axis.^23^ Initially, it was believed that Wnt3a regulated the β‐catenin and YAP/TAZ through the same mechanism. A recent study, however, refuted this idea, arguing that while Wnt3a can activate YAP/TAZ and β‐catenin concurrently, their activations use different mechanisms.^18^, ^24^

Conversely, YAP/TAZ influences the Wnt signaling pathway. By interacting with the β‐catenin destruction complex, YAP/TAZ can regulate Wnt signaling.^25^ Inducers such as SOX2, lysophosphatidic acid (LPA), Ga12/13, and RhoA activate YAP/TAZ. Under the influence of LPR6, YAP1 not only combines with β‐catenin, but also directly induces the expression of dickkopf‐1 (Dkk1), an antagonist to Wnt signaling. This ultimately regulates the differentiation of osteoblasts.^26^ Additionally, a study demonstrated that YAP/TAZ can interact with β‐catenin on SRY‐related HMG box (Sox2) and snail homolog 2 (Snai2) genes, leading to inhibition of the Wnt signaling pathway. This interaction prevents myocardial cell proliferation and regulates heart size.^27^

In summary, the regulation between YAP/TAZ and Wnt signaling is bidirectional, and their interplay has significant implications for normal bone tissue growth, gene expression, and malignancies. This offers brand‐new treatment targets for a range of illnesses.^28^, ^29^

### 2.3 YAP/TAZ regulated by G protein‐coupled receptor signaling

G protein‐coupled receptors (GPCRs) signaling also plays a core role in the regulation of YAP/TAZ, which successfully prevents the phosphorylation of the YAP/TAZ. GPCRs, also known as 7 transmembrane receptors, have either ion channel structures or enzyme activity.^30^ They transmit signals through a complex cascade involving a series of signaling proteins. The effects of GPCR signaling on YAP/TAZ vary depending on ligands, which can be neurotransmitters or hormones.^31^

Sphingosine‐1‐phosphate (S1P) has different effects on YAP and LATS1/2. S1P enhances Rho GTPases to promote YAP activity, while it suppresses LATS1/2 activity through its interaction with GPCRs.^32^, ^33^ Similarly, protease‐activated receptor‐1 (PAR1), which participates in the invasion and transfer of cancer cells, can also inhibit LATS1/2 through Rho GTPases to activate YAP/TAZ, finally promoting epithelial–mesenchymal transition (EMT).^34^, ^35^ Notably, the GPCR PAR1 can be regulated by twist and α‐arrestin domain containing protein 3 (ARRDC3) and ARRDC3 is able to inhibit TAZ, which plays a role in blocking PAR1 to come into play.^35^, ^36^ Moreover, angiotensin II, which binds to the angiotensin II type 1 receptor (AT1R), a GPCR, causes the unphosphorylated YAP to shuttle increasingly between the nucleus and the cytoplasm, facilitating cell apoptosis upon reactivation.^37^ Lysophosphatidic acid (LPA) is a lipid mediator that can influence the cell proliferation, differentiation, and migration and LPA can combine with six GPCRs (LPA1–LPA6) at least to play a role.^38^ LPA4 and LPA6 can couple to Ga12/13 to activate the Rho‐ROCK signaling pathway, promoting YAP/TAZ expression and inhibiting β‐catenin/Notch intracellular domain (NICD)‐induced activation of angiogenesis factor delta‐like ligand 4.^39^, ^40^ Similarly, thromboxane A2 and its GPCR TP can also upregulate YAP/TAZ by coupling to Ga.^41^ In addition, multiple metabolites (like fatty acids, bile acids, purines, and adenosine) activate YAP by binding to GPCR.^42^ However, various ligands had been discovered to work in conjunction with diverse GPCRs to activate YAP/TAZ to cause different diseases through the induction of Rho GTPases, including organ fibrosis, oral squamous cell carcinoma, and hepatocellular carcinoma (HCC).^34^, ^43^, ^44^, ^45^

In addition to the mediation of Rho GTPases, GPCR can also regulate YAP/TAZ through different mechanisms. The β‐adrenergic receptor (β‐AR) is also a typical GPCR, which can promote cell proliferation and lead to cancer.^46^ β‐AR is able to activate the tyrosine kinase Src and then phosphorylate YAP at tyrosine and promote cell proliferation.^47^ Besides, GPCR has been discovered to influence G12/13, Gi/o, and Gq. The activation of YAP/TAZ can be induced by Gq while independent from G12/13.^48^ Interestingly, insulin/insulin‐like growth factor 1 (IGF‐1) receptors and GPCR have a crosstalk and this crosstalk has an influence on the activation of YAP/TAZ, which finally has an impact on the secretion of insulin and the development of pancreatic cancer.^49^

To sum up, identifying the association between GPCR and the YAP/TAZ may provide insights into the mechanisms of disease development and new targets for cancer and tissue fibrosis treatment.

### 2.4 YAP/TAZ regulated by estrogen signaling

Several studies have found that YAP/TAZ exhibits crosstalk with the estrogen/ER signaling pathway, and that they regulate each other reciprocally.

On the one hand, estrogen is able to bind to G protein‐coupled estrogen receptor (GPER) to regulate the activation of YAP/TAZ. Cheng et al.^50^ demonstrated that the knock‐down of GPER can decrease YAP activation, indicating that GPER may be involved in YAP activation and subsequent upregulation of the expression of angiopoietin‐like 4, which in turn promotes cell invasion. GPER also accelerates YAP activation and nuclear accumulation in chondrocytes, blocking the RhoA/LIMK/cofilin signaling pathway in the downstream of YAP and preventing actin polymerization.^51^ In addition to activating YAP, GPER can also result in YAP inhibition. In hepatic stellate cells (HSCs), YAP expression shows a negative correlation with GPER, and the GPER/RhoA/myosin signaling pathway, activated by the GPER agonist tamoxifen, inhibits YAP activation, affecting the secretion of hypoxia‐inducible factor‐1 alpha (HIF‐1α) and extracellular matrix (ECM) proteins.^34^, ^52^

On the other hand, estrogen binding to estrogen receptors (ER) also plays an essential role in regulating YAP/TAZ activation.^53^ YAP/TEAD serve as necessary cofactors of ER.^53^ In HCC, it has been proven that the ERα makes an effect on triggering the Hippo pathway and phosphorylating YAP/TAZ, contributing to inhibiting the nuclear translocation of YAP/TAZ.^54^ However, in a mouse model, ERα activation by ginsenoside Rg1 increases the expression of YAP protein, thus attenuating the liver hepatic ischemia–reperfusion injury.^55^, ^56^ In addition to the liver, estrogen is also a vital regulatory hormone in the ovaries. Using estrogen in ovariectomized mice upregulates the phosphorylation of YAP in a time‐dependent manner, then decreases the YAP‐targeted genes expression in endometrial cells.^57^ Moreover, ERα also plays a role in regulating Src‐induced YAP activation. In ERα overexpression MCF‐7 cell, the upregulated ERα can phosphorylate YAP at Tyr357 and then enhance the expression of its downstream gene connective tissue growth factor (CTGF) through enabling Src tyrosine kinase.^58^ The mechanism by which estrogen regulates YAP is still not fully understood, but it may involve epigenetic modifications. Estrogen binding to endothelial cells positively affects the induction of recombinant DNA methyltransferase 3B (DNMT3B)‐mediated methylation of the YAP1 promoter, thereby promoting the overexpression of YAP.^59^ Additionally, a study found that the use of ER inhibitors and the Wnt/β‐catenin pathway inhibitors significantly suppressed the role of Icariin in promoting TAZ expression, suggesting that ER and Wnt/β‐catenin all participate in Icariin‐induced TAZ activation.^60^ Notably, arylsulfatase D (ARSD), predominantly expressed in females, can serve as a crucial factor regulating ERα‐mediated YAP/TAZ activation.^61^ As the downstream signal of ERα, ARSD is able to restrain YAP/TAZ activation and cancer cell proliferation through acting on the Hippo signaling pathway, implying that ARSD can be a potential target in breast cancer.^62^

Furthermore, YAP/TAZ also has an impact on the transcriptional level of ERα. The nuclear translocation of YAP can compete with ERα for the combination of TEAD to promote ERα degradation.^63^ Besides, YAP/TAZ can regulate the expression of Vestigial‐Like Protein 3 (VGLL3), while VGLL3 is able to inhibit the interaction of YAP/TAZ and TEAD in turn, which then facilitates the accumulation of nuclear receptor c0‐repressor 2 (NCOR2/SMRT) repressor to the super‐enhancer of ESR1 gene, finally resulting in ERα transcription level decrease.^64^ It should be noted that the effect of YAP inhibiting ERα transcription level may enhance the resistance of tamoxifen, thereby diminishing its normal pharmacological action.^65^

In actuality, the ER interacts with the YAP/TAZ, which is crucial in bone remodeling and breast cancer.^66^ Therefore, more research is needed to fully understand the connection between YAP/TAZ and estrogen/ER, which may also lead to new cancer treatment and bone regeneration strategies

### 2.5 YAP/TAZ regulated by mechanical signaling

However, as an important mechanical transducer, YAP/TAZ can be affected by mechanical signaling. A study showed that YAP/TAZ was sensitive to a wide range of mechanical signaling inputs, including extracellular matrix (ECM) rigidity, cell shape, and focal adhesions (FAs).^67^ These mechanical signals make an effect on YAP/TAZ, the extracellular signal is transformed into an intracellular signal by YAP/TAZ, contributing to cell growth, cell differentiation, and specific transcription.^68^ Interestingly, there are two approaches to modulating mechanical signaling to YAP/TAZ: the Hippo‐dependent way and the Hippo‐independent way.

In the Hippo‐dependent way, integrin‐containing FAs connect the ECM to the cytoskeleton via actin, forming a mechanical conduction pathway.^69^ Since the β1‐integrin mediates the activation of FAs kinase in response to ECM rigidity or low cell density, the activated FAK (FAs kinase) promotes LATS1/2 inhibition and the nuclear translocation of YAP/TAZ through the β1‐integrin–FA kinase–SRC–phosphatidylinositol 4,5‐bisphosphate 3‐kinase (PI3K)−3‐phosphoinositide‐dependent kinase 1 (PDK1) signaling pathway or the β1‐integrin–FAK–P130–Cas–Rac1–PAK‐Merlin pathway.^69^, ^70^, ^71^, ^72^ Interestingly, Cerebral cavernous malformations 3 is able to compete with FAK, thereby influencing FAK‐induced YAP/TAZ activation.^73^ In fact, a stretch, which parallels with major axis of cell, has the ability to activate YAP while loses this ability when perpendicular to major axis of cell. The paralleled stretch may lead to actin rupture and restoration, which is mediated by the FA protein zyxin and then affects nuclear translocation of YAP induced by FAK.^74^ Multiple factors upstream of integrin regulate its activation and YAP activation. Agrin, an ECM proteoglycan, can be regulated by ECM rigidity.^75^ Agrin promotes YAP activation through two ways. First, agrin combines with lipoprotein‐related receptor‐4 (Lrp4) and muscle‐specific kinase (MuSK) to influence actin stress fibers. Second, agrin promotes the integrin/FAK/ILK/PAK1 signaling pathway to inhibit the Hippo signaling pathway. In these two ways, agrin accelerates YAP nuclear translocation, disrupts the interaction between YAP and 14‐3‐3, and enhances YAP response to mechanical signaling.^76^ In addition to agrin, thrombospondin‐1, an extracellular mediator of mechanical signaling, is also beneficial in the activation of integrin/YAP pathway.^77^ Furthermore, the lower ECM rigidity promotes the expression of phosphatidylinositol 4,5‐bisphosphate and its product phosphatidic acid mediated by phospholipase Cγ1 to promote PDZGEF1/2‐induced RAP2 activation and the activated RAP2 can interact with MAP4K4/6/7 to positively affect Hippo signaling pathway and inhibit YAP/TAZ.^78^ On the contrary, the adherens junction (AJ) mediates intercellular adhesion containing α‐catenin and β‐catenin, forming a mechanical connection to actin. The α‐catenin and β‐catenin can bind to Merlin to activate the Hippo pathway and eventually impose restrictions on the nuclear translocation of YAP/TAZ.^79^, ^80^

As for the Hippo‐independent way, talin and the linker of the nucleoskeleton and cytoskeleton complex have been shown to play a crucial role in establishing mechanical connections between the cell nucleus and stress fibers.^81^, ^82^ Under a stiff substrate, force signals are transmitted to the nucleus through mechanical connections, thereby flattening the nucleus in favor of the nuclear translocation of YAP/TAZ.^83^, ^84^ Actually, in high cell density, the tensile strength of stress fibers can be increased. The increased stretching force contributes to separating Merlin from AJ, and the untrammeled Merlin has the ability to bind to YAP as a complex to inhibit YAP/TAZ activation.^85^ Through applying one of the super‐resolution imaging techniques dSTORM, the response of YAP to mechanical signaling can be observed. Cell surface pressure leads to the depolymerization of F‐actin, the downregulation of RhoA and LPAR1 and the mechanical signaling from cell surface pressure can transmit from cytoplasm to cell nucleus participated by cytoskeleton to promote the cytoplasm translocation of YAP.^86^, ^87^ Interestingly, in high mechanical stress, the F‐actin have a competition with YAP/TAZ in the combination of ARID1A/SWI/SNF, which has a positive influence on YAP/TAZ nuclear translocation.^88^ Besides, in high ECM rigidity, F‐actin can cooperate with Fascin1 to activate YAP/TAZ, while in soft ECM, the regulators of F‐actin, CAPZ, and Arp2/3 can promote the transfer of YAP/TAZ from cell nucleus to cytoplasm.^89^ Piezo is a kind of biological force‐sensing systems, which is a key factor in the response of YAP/TAZ in mechanical signaling.^90^ Actually, in different organs, piezo has diverse mechanisms in regulating YAP/TAZ. In heart valve, piezo can simultaneously regulate YAP1 in smooth muscle progenitor cells and Klf2‐Notch signaling in endothelial cells in the induction of mechanical force.^91^ And in bone, piezo is able to respond to fluid shear stress and ECM stiffness signals to activate NFAT/YAP1/β‐catenin pathway in a Ca^2+^‐dependent manner.^92^

The functions of YAP/TAZ in mechanical transduction are still being researched at this time. Targeting the mechanical connections within the mechanical transduction pathway offers tremendous potential for the treatment or prevention of cancers because this system is involved in a number of cancers by encouraging cell proliferation.

### 2.6 YAP/TAZ regulated by metabolism

Metabolism exerts a crucial influence on activity of the YAP/TAZ. Glucose and lipid metabolism participate in the regulation of YAP/TAZ, and conversely, YAP/TAZ also impacts their metabolism. Glucose can induce O‐GlcNAcylated of YAP at serine 109 by O‐linked β‐N‐acetylglucosamine (O‐GlcNAc) transferase, which hinders the interaction between the YAP and the LATS1/2 complex. This prevents LATS1/2 from phosphorylating and inhibiting the YAP, resulting in pancreatic tumorigenesis.^93^ Additionally, β‐transducin repeat‐containing protein (βTrCP), which is a ubiquitin E3 ligase mediating the degradation of YAP, loses the ability to recognize YAP through O‐GlcNAcylated YAP, thereby ensuring YAP stability.^94^ Normal aerobic glycolysis increases the transcription activity of the YAP/TAZ, whereas suppressed aerobic glycolysis has the reverse effect.^95^ The phosphofructokinase (PFK1), which catalyzes the phosphorylation of fructose‐6‐phosphate in glycolysis, can increase the activity of the YAP by combining with TEAD, and then promotes the tissue overgrowth and tumor development.^96^

Lipids and their metabolites likewise have the ability to regulate YAP, contributing to a variety of diseases. As a factor in the production of monounsaturated fatty acid production, the stearoyl‐CoA‐desaturase 1 (SCD1) contributes to maintaining the stability of YAP and promoting the activity of β‐catenin.^97^ Similar to S1P, the geranylgeranyl pyrophosphate (GGPP) from the Sterol Response Element Binding Protein (SREBP)/Mevalonate pathway was associated with the activation of the Rho GTPases, which in turn contribute to activating YAP/TAZ. The Mevalonate‐YAP/TAZ axis drives the acceleration of cell proliferation in breast cancer.^98^ Additionally, the 16‐carbon fatty acid palmitate is able to connect with the cysteine residues in TEAD, creating ideal circumstances for the fusion of YAP and TEAD.^99^

Mediated by the multiple signaling pathways mentioned above, YAP/TAZ can play a role in the emergence of a range of diseases, including inflammatory disorders, malignancies, and organ fibrosis. Therefore, by exploring the mechanism of YAP/TAZ in diseases, it can be used as a potential therapeutic target. Furthermore, based on its role in promoting cell proliferation, YAP/TAZ could also be used for organ regeneration after organ damage.

### 2.7 The epigenetic regulation of YAP/TAZ

In multicellular organisms, cells are homogeneous in terms of genes but heterogeneous in phenotypes. The heterogeneity of this phenotype does not depend on changes in DNA sequences and is heritable, affecting structure and function, which is called epigenetic regulation.^100^ Epigenetic regulation mainly includes DNA methylation, histone modification and noncoding ribonucleic acid regulation.^101^, ^102^ DNA methylation is able to change DNA conformation and chromosome structure through DNMT1, DNMT3a and DNMT3b to regulate gene expression.^103^ The histone modifications include methylation and demethylation, acetylation and deacetylation, phosphorylation and dephosphorylation, ubiquitinization and deubiquitinization. And these histone modifications can occur in gene promoters and enhancer to affect gene expression.^104^ Furthermore, noncoding RNA can also determine the direction of gene expression by influencing transcription and translation.^105^ In fact, epigenetic regulation has been proven to be closely related to tumors, cardiovascular diseases, autoimmune diseases, and metabolic syndromes.^106^ In recent years, it has been found that the expression of YAP/TAZ can also be regulated by epigenetics and plays an important role in various diseases.

A study found that YAP promoter methylation can effectively reduce the expression of YAP protein by extracting the genomic DNA of breast cancer patients who are not genetically related and using methylation‐specific polymerase chain reaction (MS‐PCR), while the level of YAP promoter methylation is related to the progress of breast cancer. The methylation level of YAP promoter in patients with highly invasive stage III and IV breast cancer is significantly higher than that of weakly invasive stage I and II breast cancer.^107^ Lysine demethylase 3A (KDM3A) is a histone H3K9me1/2 demethylase. The study found that the high expression of histone H3K9me2 on YAP can significantly reduce the YAP protein, and whether the use of demethylase KDM3A or H3K9me2 inhibitors BIX‐01294 and UNC0631 can effectively increase the expression of YAP1 in cells. YAP1 is considered to be a carcinogen, and the high expression of KDM3A‐mediated YAP1 may lead to the occurrence of colon cancer tumors.^108^ The coactivator‐associated arginine methyltransferase (CARM1) is able to induce the methylation of histone H3 arginine 17/26 to H3R17/26me. Interestingly, Yang et al.^109^ found that a large number of coactivator‐associated arginine methyltransferase 1 (CARM1)‐mediated histone H3 arginine17 methylation appeared at the transcription starting point of YAP1 during embryonic development to regulate the progress and speed of embryonic development. In addition, a large number of noncoding RNAs can also directly target YAP to regulate its epigenetic regulation, including microRNA and cyclic RNA. Li et al.^110^ found that YAP1 can play a role as a direct target for miR‐484 in the reduction of ovarian reserves. Specifically, miR‐484 directly binds to 3′ UTR on YAP mRNA in granulosa cells to lower the expression of YAP1, thus weakening the effect of YAP1 in inhibiting mitochondrial‐dependent cell apoptosis and reducing ovarian reserves.^110^ Similar to miR‐484, miR‐506 and miR‐622 can also directly target the 3′ UTR region on YAP to negatively adjust the expression of YAP.^111^, ^112^ In addition to miRNA, YAP circular RNA has also been shown to bind to YAP mRNA and translation initiation protein, thus reducing YAP expression.^113^

Unlike the epigenetic regulation of YAP, there are many mechanisms. The main people involved in the epigenetic regulation of TAZ are noncoding RNA. In fact, different miRNAs can combine with 3′ UTR on TAZ mRNA to inhibit TAZ translation and play different effects in different organs. Compared with young pulp tissue, an up–up adjustment of miR‐584 expression and a downmediated TAZ expression have been found in aging pulp tissue, which inhibits the proliferation of pulp stem cells and affects pulp regeneration.^114^ In the ovaries, miR‐125a‐5p has a complementary binding region with 3′ UTR on TAZ, which inhibits the expression of TAZ and ultimately reduces the epithelial–mesenchymal transformation of cancer cells in the ovary.^115^ Interestingly, cyclic RNA can participate in regulating the binding of miRNA and TAZ. Cyclic RNA circ_0000511 and has_circ_0091074 have been proved to serve “sponges” of miRNA to reverse the reduction of miRNA‐mediated TAZ expression by directly targeting miRNA.^116^, ^117^

In short, epigenetic regulation is an important part of the regulation of YAP/TAZ and mediates the occurrence and development of many diseases. Targeting epigenetic regulation of YAP/TAZ may become a new method for the treatment of some diseases.

### 2.8 The posttranslational modifications of YAP/TAZ

Epigenetic regulation is the regulation of YAP/TAZ at the genetic level, which belongs to pretranslation regulation. In fact, posttranslational modification has also been found to affect the activity or function of YAP/TAZ, and this regulation belongs to the regulation at the protein level. The posttranslational modification of YAP/TAZ covers a wide range, including phosphorylation, acetylation, methylation, and O‐GlcNAcylation.^118^

As mentioned above, phosphorylation and nonphosphorylation of YAP/TAZ are the most common forms of their function. Phosphorylated YAP/TAZ is limited to the cytoplasm and degraded, while phosphorylated YAP/TAZ can undergo nuclear translocation and bind to TEAD in the nucleus. The phosphorylation of YAP/TAZ can be divided into Hippo‐dependent way and Hippo nondependent ways. For the Hippo‐dependent way, the activated LATS1/2 directly targets YAP/TAZ to phosphorylate and inactivate it.^64^ For the Hippo nondependent ways, YAP/TAZ can serve as a target of multiple molecules, such as AMP‐activated Protein Kinase (AMPK), β‐AR, and mTORC2.^47^, ^119^, ^120^ Under the action of these molecules, YAP/TAZ is inhibited or activated, which affects inflammation, cancer, metabolism, organ fibrosis, and organ regeneration. Interestingly, the phosphorylation of YAP/TAZ is not only located at one site. On the one hand, threonine and serine S127, S352, S274, S381 on YAP and S89/311 on TAZ can be phosphorylated so that YAP/TAZ can accumulate in the cytoplasm.^121^, ^122^, ^123^ On the other hand, the latest research shows that in biliary epithelial cells, serotonin (5‐HT) plays an important role in activating YAP by phosphating tyrosine on YAP.^124^

Acetylation and deacetylation of YAP also significantly affect its activity. Under the action of nuclear acetyltransferases CREB binding protein (CBP) and p300, the Lys‐494 and Lys‐497 at the C‐terminal of YAP can be acetylated. And this acetylation is conducive to the nuclear translocation of YAP.^125^ In contrast to CBP and P300, SIRT1 plays a role in the deacetylation of YAP at Lys 494, which can transfer YAP from the nucleus to the cytoplasm and promote YAP degradation. At the same time, the SIRT1‐mediated YAP deacetylation can reverse the effect of CBP and P300 on YAP.^126^ Interestingly, in the liver, SIRT1‐mediated YAP deacetylation can be regulated by pregnane X receptor, and in human A549 cells, P53 can inhibit SIRT1 to enhance the acetylation of YAP.^127^, ^128^

More and more studies have found that the methylation of YAP can regulate its biological function. Fang et al.^129^ proved that lysine methyltransferase SET1A can methylate YAP at K342 with the participation of PPxY on the YAP WW domain. Under the action of SET1A, the transcriptional activity of YAP/TEAD is improved, while blocking the transfer of YAP to cytoplasm.^129^ The YAP methylation mediated by SET1A can be inhibited by the lipolytic factor a/b‐hydrolase domain‐containing 5 (ABHD5). ABHD5 can locate DPY30, which is the core subset of SET1A methyltransferase in the cytoplasm, and undergo ubiquitination and degradation of YAP.^130^ In addition to SET1A, the methylation of YAP can also be induced by suppressor of variegation 3‐9‐enhancer of zeste‐trithorax domain containing lysin E methyltransferase 7 (SETD7). SETD7 can methylate the YAP at lysine 494 to inhibit the transfer of YAP to the nucleus, thus reducing the transcription of antioxidant genes manganese superoxide dismutase (MnSOD) and catalase (CAT) ultimately to protect the heart.^131^ Spectrin beta, nonerythrocytic 1 (SPTBN1) can promote the expression of SETD7 to enhance YAP methylation, and ultimately plays a role in cell autophagy.^132^

The O‐GlcNAcylation of YAP has been mentioned above. In a high‐sugar environment, O‐linked β‐N‐acetylglucosamine (O‐GlcNAc) transferase (OGT) can promote O‐GlcNAcylation of YAP at Ser109, thus improving the expression of YAP1. The O‐GlcNAcylated YAP1 can further phosphorylate Akt and inhibit the expression of GSK1β.^133^ The von Hippel‐Lindau tumor suppressor protein (pVHL) is an important part of E3 ubiquitin ligase. Hu et al. found that the upregulation of pVHL expression can reduce the lysosome degradation‐dependent YAP transcriptional activity and protein level. Interestingly, using OGT can reverse the pVHL‐mediated YAP reduction, indicating that a high level of O‐GlcNAcylation can effectively protect YAP from pVHL damage and thus hinder the tumor inhibition effect of pVHL.^134^ Corosolic acid (CA) is a hypoglycemic drug with remarkable effect. Studies have shown that CA can reduce the O‐GlcNAcylation of YAP by inhibiting cyclin‐dependent kinase 19 (CDK19), which means that CA may reverse the function of O‐GlcNAcylated YAP on the tumor inhibition effect of pVHL.^135^ It is worth noting that the O‐GlcNAcylation of YAP at Thr24 can improve the expression of transferrin receptor, thus increasing the sensitivity of HCC cells to iron death.^136^

In fact, in addition to YAP/TAZ being modified after translation, its upstream or downstream signals can also be modified after translation to adjust the function of YAP/TAZ. For example, geranylgeraniol can be transformed into GGPP after entering the cell, and GGPP can stimulate the conversion of GDP on RhoA to GTP to enhance the activity of RhoA. The activated RhoA further promotes the transfer of YAP in the cytoplasm to the nucleus.^137^ However, this process can be inhibited by statins.^138^ In addition, TEAD can occur auto‐palmitoylation, through connecting the 16‐carbon palmityl group to the cysteine residue of TEAD through thioester bonds. The ability of TEAD to interact with YAP is enhanced after auto‐palmitoylation, and its own transcriptional activity is also improved.^139^ At present, TEAD auto‐palmitoylation inhibitors are being widely studied in order to block the effect of YAP/TEAD.^140^ In short, the posttranslational modification of YAP/TAZ deserves widespread attention, which may improve new ideas for the treatment of diseases.

## 3 METABOLISM REGULATED BY YAP/TAZ

As a matter of fact, the YAP/TAZ has an effect on the regulation of metabolism in turn, suggesting that the YAP/TAZ can modulate nutrition and metabolism‐related diseases.

### 3.1 Glucose metabolism

The YAP/TAZ makes an impact on regulating the enzymes and protein of glucose synthesis, glucose transport, and glucolysis. The carbohydrate response element binding protein (ChREBP) is a transcription factor. Since its deletion leads to inhibition of the glycolytic pathway and accelerates glycogen accumulation, it becomes a significant factor in glucose metabolism.^141^ In the cell nucleus, YAP/TAZ binds to ChREBP and contributes significantly to the glycolytic pathway. The phosphorylation and inhibition of the YAP/TAZ by the active Hippo pathway, as well as the subsequent suppression of ChREBP transcriptional activity, all have a detrimental effect on glucose utilization.^142^

YAP has a valuable influence on getting command of the gene expression and the enzymatic activity of the glycolytic pathway and gluconeogenesis. Blood glucose level depends on the balance of insulin and glucagon, and YAP lies at the core position in this balance.^143^ In osteosarcoma, the increased expressions of S1P and its receptor S1PR3 inhibit the phosphorylation of YAP, promote the binding of YAP and cMYC, and finally enhance the transcription of recombinant phosphoglycerate mutase 1 (PGAM1), which is a significant enzyme in aerobic glycolysis.^32^ Additionally, in cardiomyocytes, the glucose transporter‐1 (GLUT1) contributes to glucose transport and its transcription is advanced through the cooperation of YAP, TMAD, and HIF‐1α so YAP may make an effect on regulating cardiomyocyte hypertrophy.^144^, ^145^ Furthermore, proliferator‐activated receptor gamma coactivator 1 (PGC1α) is also a target of YAP. Under the function of YAP, PGC1α loses the ability to bind to the promoters of its gluconeogenic targets, and so its transcription is inhibited.^146^ Recently, a study proposed that long noncoding RNA (lncRNA) binding to the Hippo pathway participates in metabolic reprogramming. YAP plays a vital role in its downstream targets lncRNA breast cancer antiestrogen resistance 4 (lncRNA BCRA4), which contributes to the transcription of two glycolysis activators, hexokinase 2 (HK2) and 6‐phosphofructo‐2‐kinase (PFKFB3), and then promotes the YAP‐dependent glycolysis in turn.^147^ Interestingly, the role of YAP in glycolysis is extensive, exerting a widespread influence on the activities of nearly all the enzymes involved in this metabolic process.^148^

TAZ, on the other hand, participates in glycolysis to a lesser extent than YAZ but is nonetheless important. In virtue of the WW domain, TAZ can collaborate with the ligand‐binding domain of glucocorticoid receptor (GR) to astrict the gluconeogenic gene promoters and glucogenesis in the liver.^149^ This contribution of TAZ helps maintain the stable glucose concentrations in the liver. Additionally, activated TAZ has been demonstrated to have an essential effect on promoting the synthesis and transcription of pancreatic and duodenal homeobox 1 (PDX1) and then increasing insulin production in pancreatic β‐cells with the result of lowering blood sugar.^150^ As a result, TAZ can therefore affect the manufacture of insulin and afterwards indirectly control blood sugar levels.

### 3.2 Amino acid metabolism

The regulation of amino acid metabolism by YAP/TAZ is being linked in research to an expanding number of illnesses. As a matter of fact, YAP/TAZ may affect amino acid metabolism in different parts of the body. In the lung, activated YAP/TAZ has been found to control amino acid metabolism through the mTORC1–ATF4 pathway.^151^ However, YAP/TAZ can also improve the activity of mTORC1 by means of enhancing the expression of the high‐affinity L‐type amino acid transporter (LAT1). Interestingly, in the liver, because YAP/TAZ is able to interact with TEAD to directly induce the transcription of SLC7A5, which is one of the subunits of the LAT1, they make an effect on increasing the ingestion of amino acids.^152^ Thus, finding the relationship between YAP/TAZ, mTORC1, and amino acid metabolism may offer a deeper understanding and new treatment targets for the metabolic disease in the lung and liver.

Beyond direct regulations, YAP/TAZ can also indirectly influence amino acid metabolism by affecting the expression of enzymes. In breast cancer cells, the scarce liver kinase B1 (LKB1) can promote the activation of YAP/TAZ and contribute to the activation of key enzymes in serine metabolism. Moreover, the substantial increase of TAZ S89A can directly promote the expression of the key enzymes, such as phosphoserine phosphatase.^153^ Glutamine is another important amino acid involved in the regulation of YAP/TAZ, and most importantly, the YAP/TAZ regulating glutamine metabolism relates to multiple diseases. ECM stiffening makes an effect on activating YAP/TAZ. The activated YAP/TAZ plays a crucial role in activating the glutaminase (GLS), ensuring that they can harmonize glutaminolysis, which means YAP/TAZ has an impact on facilitating vascular cell growth.^154^ In addition, YAP/TAZ can induce the expression of glutamic‐oxaloacetic transaminase (GOT1) and phosphoserine aminotransferase (PSAT1), which can mediate the formation of α‐ketoglutarate (AKG) through the transamination of glutamate.^155^ Furthermore, another study proposed a different mechanism of YAP/TAZ influencing glutamine metabolism. This study demonstrated that YAP/TAZ promotes glutamine metabolism by means of differential transcriptional regulation of SLC1A5 and GLS, which are associated with glutamine decomposition.^156^

In brief, since YAP/TAZ had been shown to regulate amino acid metabolism in cirrhosis, breast cancer, and pulmonary hypertension, they may be the therapeutic targets of these diseases.^157^ Therefore, the mechanisms of YAP/TAZ in these disorders must therefore be further investigated in order to definitively establish the upstream signaling and downstream targets.

### 3.3 Lipid and nucleotide metabolism

However, in addition to glucose and amino acid metabolism, YAP/TAZ also influences lipid and nucleotide metabolism. Recently, the role of YAP/TAZ in lipid and nucleotide regulation has been the subject of ongoing study. The dysfunction of YAP, which is essential for maintaining healthy fatty acid oxidation, can lead to metabolic disorders. In obese insulin‐resistant individuals, impaired YAP function leads to incomplete fatty acid oxidation, resulting in lipotoxicity in adult skeletal muscle.^158^ Interestingly, the overexpression of YAP may lead the cancer cells to become more sensitive to ferroptosis.^159^ Researchers investigating the connection between YAP and ferroptosis revealed an unexpected finding: transcriptional upregulation of the arachidonate lipoxygenase 3 (ALOXE3) through the regulation of YAP led to the accumulation of lipid peroxides.^160^ In addition, YAP/TAZ may interact with TEAD1 to regulate the activation of uncoupling protein 1 in brown adipocytes and the mechanical sensitivity of YAP/TAZ may influence thermogenic activity in adipocytes.^161^

YAP/TAZ is essential for sustaining nucleotide synthesis, which supports the growth and development of organs. YAP has been found to be necessary for the consumption and utilization of glucose in de novo nucleotide synthesis by studies on YAP mutant and transgenic zebrafish.^145^ Similarly, YAP not only enhances the expression and activity of glutamine synthetase (GLUL) but also reprograms nitrogen metabolism to stabilize nucleotide biosynthesis.^162^ However, the latter condition may cause hepatomegaly and even liver tumors, suggesting that YAP also has a negative influence so we should approach the role of YAP in this situation dialectically.

**FIGURE 3:**
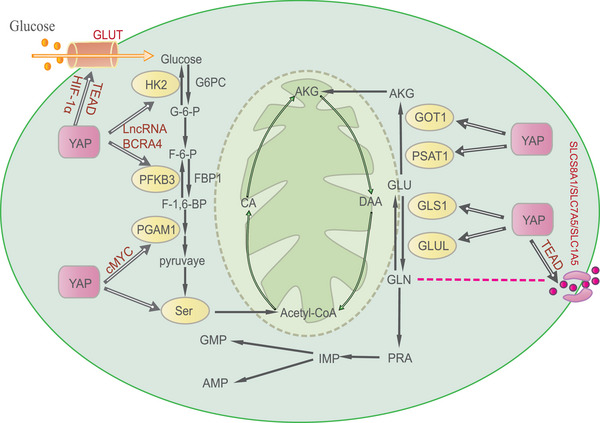
The mechanisms of YAP in metabolism. YAP plays a significant role in glucose, amino acid, and nucleotide metabolism. In glucose metabolism, not only glucose transporters but also enzymes in glycolysis can be regulated by YAP directly or indirectly. And YAP can also modulate amino acid metabolism by regulating transporters. As for glutamic acid (Glu), in one hand, YAP promotes its transformation into AKG, which is a part of tricarboxylic acid cycle through activating GOT1 and PSAT1. In another, YAP can also modulate GLUL to convert Glu to glutamine (Gln), thereby entering nucleotide synthesis.

In conclusion, YAP/TAZ plays a role in regulating the process of metabolism and it is involved in influencing the activity of various enzymes (Figure 3). Therefore, YAP/TAZ may be a promising candidate for the treatment of disorders associated with metabolism, such as metabolic syndrome, diabetes, and liver conditions.

## 4 YAP/TAZ AND CANCER

More and more research indicates that YAP/TAZ is crucial to the growth of cancer. Cell proliferation can be mediated by YAP/TAZ, however when YAP/TAZ is overactive, cancer can result from unchecked cell growth. In addition, cancer cells with high expression of YAP/TAZ can develop resistance to cancer drugs, thus hindering chemotherapy for cancer.

### 4.1 Liver cancer

There are several subtypes of liver cancer, including HCC, cholangiocarcinoma (CC), and hepatoblastoma, what is more, the incorporation of HCC and CC had also been found.^163^ Interestingly, more and more pieces of evidence showed that YAP/TAZ played an important role in the emergence of liver cancer.^164^ About 60% of liver cancer patients have been shown to express the YAP/TAZ gene, and a number of upstream signals are involved in YAP/TAZ activation.

Under the function of USP10, which is a kind of deubiquitinating enzyme, the stability of the YAP/TAZ is strengthened, accelerating the HCC's proliferation.^165^ Besides, in an MYC and β‐catenin activated rat model, the activity of YAP/TAZ also has a significant enhancement and results in the uncontrollable proliferation of liver cancer cell.^166^ Actually, except for the direct influence of the liver cancer cell biological behavior, YAP/TAZ can also decrease the sensitivity of liver cancer cell to anticancer drugs. Verteporfin (VP) is a classic inhibitor of YAP/TAZ and it had been used in the treatment of HCC.^167^ However, the ability of VP to enter into the liver cancer cell can be disrupted when YAP/TAZ has a high expression.^168^ Additionally, through the interaction between YAP/TAZ and ATF4, HCC cells are prone to develop drug resistance to another anticancer drug Sorafenib.^169^ In a word, not only the activation of YAP/TAZ but also their effect on the anticancer drugs promotes the development of liver cancer. The different mechanisms of YAP/TAZ in aggravating liver cancer also bring diverse ideas for the treatment of HCC: inhibit the expression of YAP/TAZ or find other drugs that can prevent from the function of YAP/TAZ.

### 4.2 Lung cancer

Small‐cell lung cancer and non‐small‐cell lung cancer (NSCLC), which includes large‐cell lung carcinomas, lung squamous cell carcinomas, and lung adenocarcinomas 170, are the two main subtypes of lung cancer.^170^ It had been discovered that YAP/TAZ encouraged the growth of lung cancer.

In lung‐tumor propagating cells, YAP/TAZ has an increased activity, indicating that YAP/TAZ may participate in the transference of lung‐tumor cells.^171^ Furthermore, acting as a tumor suppressor, transforming growth factor‐β (TGF‐β) receptor 2 (TGFBR2) has the ability to inhibit the development and growth of lung tumor. However, YAP/TAZ makes a contribution to promoting the evolution of lung cancer through decreasing the expression of TGFBR2 and YAP/TAZ and enhancer of zeste homolog 2 (EZH2) have a synergistic effect in this process.^172^ In addition, there is a positive feedback loop in the process of YAP/TAZ regulating lung cancer. The deficiency of Surfactant protein A1 (SFTA1P) plays a crucial role in NSCLC cell proliferation and programmed cell death. YAP/TAZ has been demonstrated that is able to increase the expression of SFTA1P through the participation of TEAD. Interestingly, the activated SFTA1P has a significant impact on promoting the expression of YAP/TAZ in turn, therefore highly promoting tumor growth.^173^ Besides, YAP also has an influence on increasing drug resistance in lung cancer. As a choice drug, cisplatin (CDDP) has been used in the treatment of lung cancer. Nevertheless, in lung‐tumor cells, the drug resistance of CDDP may be increased by the overexpression of YAP.^174^ Except for CDDP, YAP serves an essential role in epidermal growth factor receptor (EGFR)‐tyrosine kinase inhibitors (TKIs) drug resistance of NSCLC. Surprisingly, gossypol had been proved that played an effective role in surmounting the EGFR‐TKIs drug resistance by inhibiting YAP/TAZ.^175^

### 4.3 Breast cancer

Approximately 170 million women worldwide are diagnosed with breast cancer annually, making it the second leading cause of death among women.^176^ Despite the fact that breast cancer primarily affects women, it is important to note that men can also be diagnosed with breast cancer, referred to as male breast cancer. Breast cancer is divided into five types based on the different expressions of ER, progesterone receptor, and ERBB2 (HER2): luminal A, luminal B, HER2, basal‐like, and triple‐negative (TNBC).^177^

YAP/TAZ exerts a significant impact on the metastasis and aberrant proliferation of breast cancer. In the nucleus, YAP has been reported to inhibit the transcription of growth differentiation factor (GDF15) and then promotes breast cancer metastasis.^178^ Expect for the GDF15, the interaction between the S127A‐mutated YAP and TEAD also has an influence on breast cancer metastasis.^179^ Additionally, the excessive expression of TAZ in low‐expressing differentiated, nontumorigenic, breast cancer cells facilitates tumor cell transportation and induction. Similarly, the metastatic colonization of breast cancer stem cells may be limited when the TAZ is deficient.^180^ Encouragingly, a study has shown that in TNBC, the inhibition of TAZ by Synaptopodin‐2 reduces cancer cell metastasis.^181^ In addition to the metastasis of breast cancer, the activated YAP/TAZ also results in the abnormal proliferation of breast cancer cells. Sperm‐associated antigen 5 (SPAG5) is a kind of oncogene in breast cancer.^182^ The transcription of SPAG5 can become unusual and cause this oncogene mutation in the function of YAP/TAZ/TEAD, leading to breast cancer cell proliferation out of control.^183^ Anticancer drug resistance is one of the major causes of death in breast cancer patients. Notably, YAP/TAZ is involved in tamoxifen‐resistant as well as trastuzumab‐resistance in breast cancer, indicating that the survival rate may increase through inhibiting the activity of YAP/TAZ.^184^, ^185^ Interestingly, tamoxifen‐resistant MCF7 breast cancer cells have been shown to restore sensitivity to anticancer drugs via the YAP/TAZ–recombinant phosphoserine aminotransferase 1 (PSAT1) axis.^185^

**FIGURE 4:**
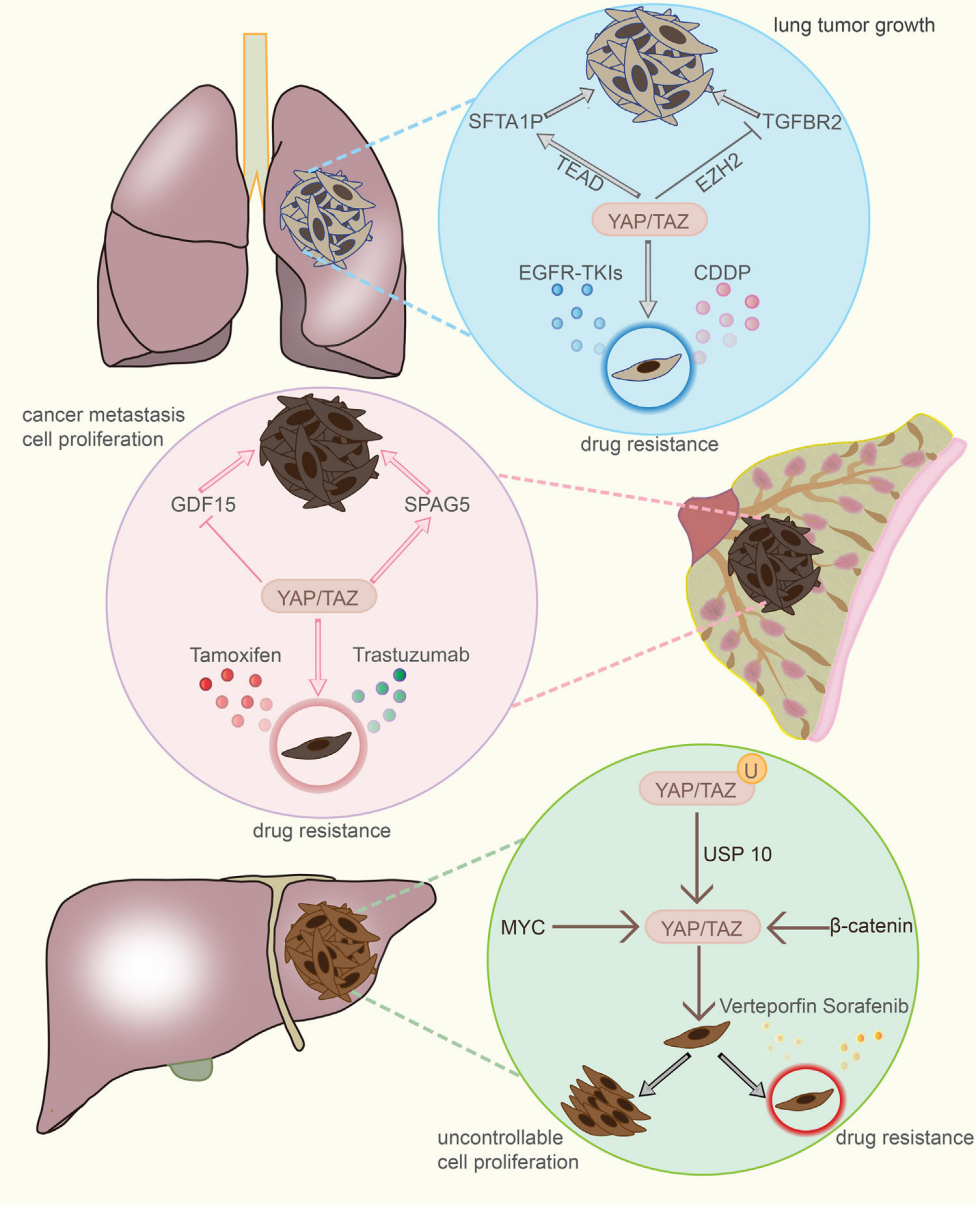
YAP/TAZ participants in cancer cell growth and anticancer drugs resistance. YAP/TAZ participants in the developments of cancers, including liver cancer, lung cancer, and breast cancer. The activation of YAP can lead to cancer cell uncontrolled proliferation and cancer cells becoming insensitive to anticancer drugs.

In conclusion, numerous investigations have shown that activated YAP/TAZ plays a crucial part in aggressive cancers. Furthermore, multiple upstream signals can induce the proliferation and diffusion of cancer cells by regulating YAP/TAZ. In addition to induce cancer metastasis and tumor proliferation, YAP/TAZ can also affect the resistance of some common anticancer drugs, leading to disease progression and even death (Figure 4). Indeed, YAP/TAZ has been implicated various types of cancers (Table 1). Therefore, targeting YAP/TAZ and its associated signaling pathways may serve as potential therapeutic strategies to inhibit the cancer cell proliferation and overcome drug resistance. Further research is warranted to design new drugs targeting YAP/TAZ.

**TABLE 1:** Summaries of the mechanism of YAP / TAZ in cancers.

Associated cancer type	Upstream signaling or downstream response	Mechanism of action	References
Colorectal cancer	Endothelin receptor A (ETAR) and G‐protein Gαq/11 and Rho GTPase	Inhibit the Hippo pathway then YAP/TAZ activate	186
	Periostin and Integrin–FAK–Src pathway	The activation of YAP/TAZ and IL‐6 expression in tumor cells	187
	SYNPO2	Regulate YAP–KLF5 axis	188
Oral squamous cell carcinoma (OSCC)	GPR39–Gαq– RhoA(ROCK)–FAK signal pathway	Yap abnormal activation	189
	PIEZO1	YAP/PIEZO1 axis promotes OSCC cell growth	55
Head and neck squamous cell carcinoma	PI3Kα–PDK1–AKT–mTOR signal pathway	Yap abnormal activation	190
	EGFR	Inhibit the Hippo pathway then YAP/TAZ activate	191
	FAT1		192
Gastric cancer	MYC	YAP/TAZ increase the expression of MYC	193
	RNA HCG18 and miR‐141‐3p	Increase expression of WIPF1 and YAP/TAZ	194
Pancreatic cancer	CS‐GRP78 and AKT kinase (AKT)/DLC1 Rho‐GTPase‐activating protein (DLC1) complex	Increase the nuclear accumulation and target gene expressions of YAP/YAZ	195
	MEKK3 (MAP3K3)	Positive regulator of YAP/TAZ	196
	Insulin and neurotensin with PANC‐1 or MiaPaCa‐2 cells	Promote YAP nuclear localization and decrease YAP phosphorylation	197
Glioma	Actin‐like 6A (ACTL6A)	Break the interaction between YAP and β‐TrCP E3 ubiquitin ligase	198
Glioblastoma multiforme (GBM)	BEX1 and BEX4	Regulation of actin polymerization and activation of YAP/TAZ signaling	199

## 5 YAP/TAZ AND ATHEROSCLEROSIS

Atherosclerosis is an inflammatory disease. Atherosclerosis is generally associated with severe cardiovascular complications. Once plaques become too large and completely block the lacuna vasorum, ischemia and infarction of the corresponding organ may happen. At the same time, atherosclerosis is prone to cause aneurysms, and the rupture of aneurysms can cause life‐threatening haemorrhages.^200^ It is now recognized that atherosclerosis is mediated by endothelial inflammation and the inflammatory macrophage. Targeted anti‐inflammatory therapy plays a crucial role in the prevention and treatment of atherosclerosis.^201^ YAP/TAZ has been shown to be a therapeutic target of atherosclerosis and YAP can both participate in endothelial cell inflammation and influence the biological behavior of macrophages in atherosclerosis. However, the mechanisms underlying the role of YAP/TAZ in the development of atherosclerosis are still being explored.

Dysregulation of NLRP3 inflammatory vesicles is involved in the development of atherosclerosis.^202^ A study demonstrated that YAP can prevent the ubiquitination of K27 at NLRP3 lys380, which is mediated by the E3 ligase β‐TrCP1and this ubiquitination leads to degradation of the NLRP3 proteasome. Therefore, through activating the Hippo signaling pathway, YAP is phosphorylated and inhibited and the activity of NLRP3 inflammatory vesicle significantly decreases.^203^ Therefore, activating Hippo signaling pathway to inhibit YAP may be a novel therapeutic idea for atherosclerosis. Additionally, YAP had been found to modify the mRNA methylation of inflammation‐related genes in endothelial cells to increase gene expression and thus exacerbate endothelial inflammation.^204^ In fact, in atherosclerosis, the effect of YAP on endothelial inflammation can be regulated by a variety of upstream signals. The transcription factor BTB and CNC homology 1 (BACH1) was found to colocalize with YAP, and BACH1 can bind to the YAP promoter to increase the expression of YAP in endothelial cells, during which the expression of proinflammatory genes and atherosclerotic plaque adhesion molecules is also upregulated.^205^ This phenomenon suggests that BACH1 can induce YAP overexpression and then promotes the development of atherosclerosis and vascular inflammation. In addition, S‐nitrosylation of guanine nucleotide‐binding protein G(i) subunit alpha‐2 (SNO‐GNAI2) was found to play a role in LATS1 dephosphorylation by coupling with C‐X‐C chemokine receptor (CXCR5) in diabetic patients, thereby blocking the Hippo signaling pathway and ultimately promoting YAP nuclear translocation and endothelial inflammatory responses.^206^ The exploration of upstream signaling in YAP‐dependent endothelial cell inflammation implies that not only YAP could be a target for atherosclerosis therapy, but also its upstream signals could be potential targets. Interestingly, blood flow status can also have an impact on YAP activity and endothelial cell inflammation in atherosclerosis. Indeed, vessels with a predominantly turbulent flow are more prone to cause atherosclerosis, while laminar flow appears to have a preventive effect on atherosclerosis.^207^ Turbulence can induce YAP/TAZ expression and nuclear translocation in endothelial cells, thereby promoting proinflammatory gene expression and monocyte adhesion. And the activated YAP/TAZ promotes that blood vessels change into atherosclerotic susceptibility phenotype.^208^ Conversely, laminar flow contributes to LATS‐mediated YAP phosphorylation in endothelial cells, thereby inhibiting YAP nuclear translocation and downregulating the expression of YAP target genes (CTGF and Cyr61).^209^ In current study, there are two main mechanisms for the protective effect of laminar flow on atherosclerosis. On the one hand, laminar flow can induce endothelial autophagy and cause YAP degradation. Laminar flow can also promote SIRT1‐mediated YAP deacetylation, which causes YAP to be transferred from the nucleus to the cytoplasm and then degraded by endothelial autophagy.^126^ On the other hand, laminar flow can act as a unidirectional shear stress to activate the integrin signaling pathway and induce integrin binding to Ga13, thereby inhibiting RhoA‐mediated YAP activation. The inactivated YAP further loses the ability to promote the transmission of its downstream signaling pathway c‐Jun N‐terminal kinase (JNK), thereby reducing inflammation.^210^ Thus, activations of the SIRT1‐mediated YAP deacetylation‐endothelial autophagy andintegrin–Ga13–RhoA–YAP–JNK signaling pathway may have a positive effect on the treatment of atherosclerosis. In addition to endothelial cells, the overexpression of YAP in macrophages can also exacerbate atherosclerosis. In YAP overexpressing macrophages, interleukin 1β (IL‐1β) can not only inhibit the degradation of YAP proteasome to promote YAP accumulation, but also break the interaction of YAP and angiomotin (AMOT), and this inhibition is mediated by TNF receptor associated factor 6 (TRAF6)‐induced ubiquitination of YAP.^211^ Specifically, this study also found that the antiatherogenic effect of the drug was diminished in YAP overexpressing macrophage of mice.^211^ Therefore, YAP overexpressing macrophages could be a potential target not only for the treatment of atherosclerosis but also for recovering the normal pharmacological effects of antiatherosclerotic drugs. Antiatherosclerotic drugs targeting YAP have been identified. Low‐dose methotrexate treatment may reduce the incidence of cardiovascular disease. Methotrexate can activate AMP‐activated protein kinase (AMPK) and then promotes AMPK‐mediated phosphorylation and inactivation of YAP at Ser127, ultimately reducing turbulence‐induced atherosclerotic plaque formation.^212^ Salicylic acid B (Sal‐B) was previously considered as a promising candidate for the treatment of Alzheimer's disease and dementia because it has anti‐inflammatory effects. A study discovered that Sal‐B can inhibit the YAP/TAZ/JNK signaling pathway, thereby reducing the production of oxidized low‐density lipoprotein (LDL) and reducing inflammation.^213^ In addition, naringin has also been considered as a potential drug for the treatment of atherosclerosis, with a similar mechanism of to that of Sal‐B.^214^

**FIGURE 5:**
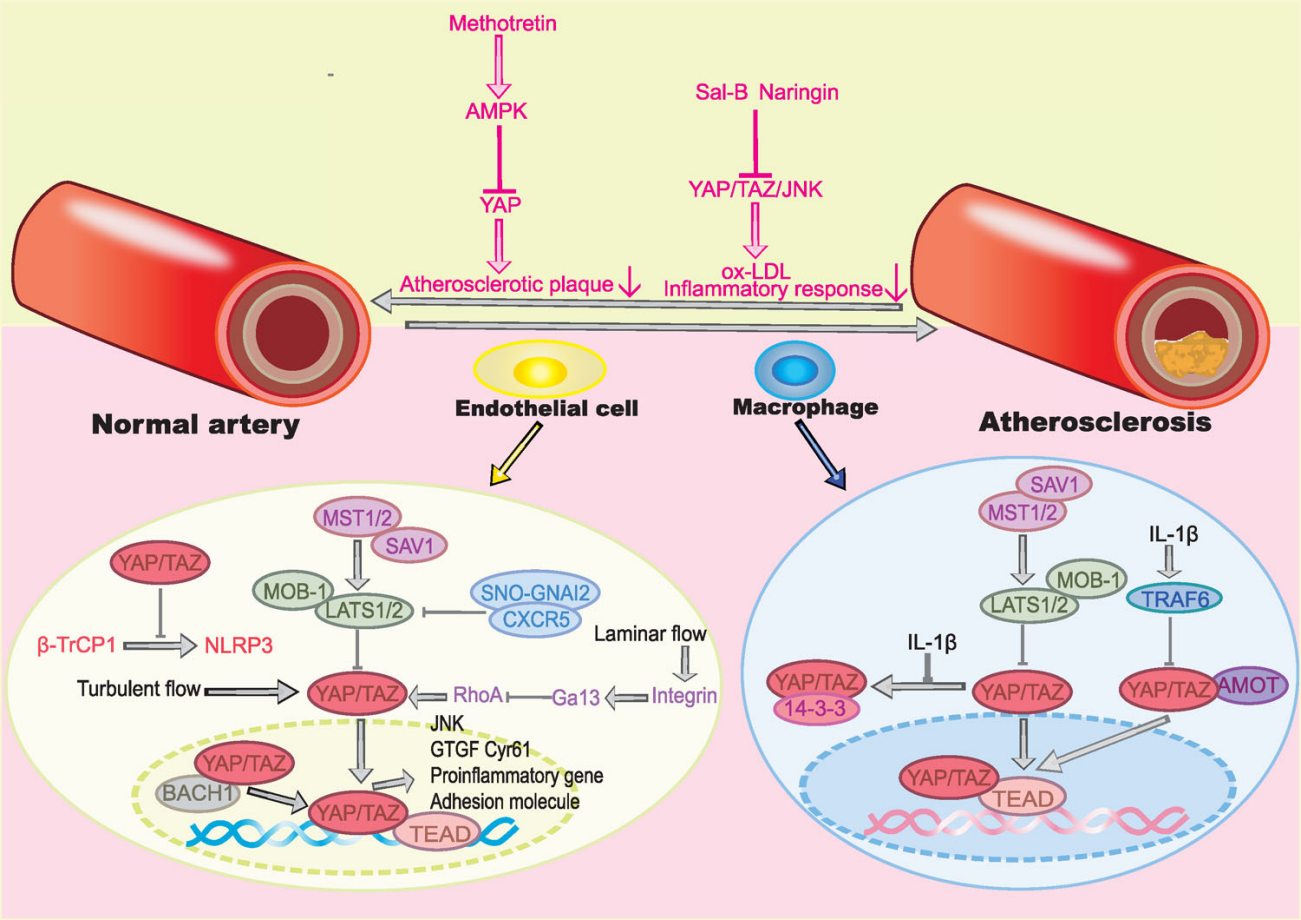
YAP/TAZ influences the transition of normal artery and atherosclerosis. In endothelial cell and macrophage, YAP/TAZ can be activated through diverse upstream signals to promote the expression of proinflammatory genes and the release of inflammatory factors, finally leading to atherosclerosis. Besides, methotretin, Sal‐B, and naringin have been demonstrated that play a role in inhibiting YAP/TAZ to improve atherosclerosis.

In conclusion, YAP/TAZ has been demonstrated to be a therapeutic target for atherosclerosis and is implicated in the formation of atherosclerosis through regulation of endothelial cells and macrophage (Figure 5). The modulation of YAP/TAZ‐induced atherosclerosis can involve a number of upstream signals. As a result, more research into the role of YAP/TAZ in atherosclerosis would be helpful in discovering more possible therapeutic targets for atherosclerosis treatment as well as novel suggestions for atherosclerosis prevention.

## 6 YAP/TAZ AND FIBROSIS

YAP/TAZ affects the biological behavior of cells. On the one hand, YAP/TAZ‐mediated cell proliferation can lead to cancer. On the other hand, YAP/TAZ can also regulate cell differentiation. YAP/TAZ can promote the differentiation of fibroblasts to muscle fibroblasts, leading to collagen deposition and organ fibrosis.

### 6.1 Myocardial fibrosis

Myocardial fibrosis is a group of cardiovascular diseases. Myocardial fibrosis is usually caused by the cardiac fibroblasts activation and further differentiation into myofibroblasts. Myofibroblasts can express α‐smooth muscle actin (α‐SMA) and secrete ECM, leading to the formation of fibrotic scarring.^215^ Prolonged fibrosis may predispose to heart failure, which can seriously affect cardiac function.^216^ Therefore, inhibiting the transdifferentiation of fibroblast into myofibroblast is of great importance in the prevention and treatment of myocardial fibrosis and in ensuring the normal function of the heart. Numerous investigations have indicated that YAP was crucial in the activation of fibroblasts and the emergence of myocardial fibrosis.^217^ As YAP had been identified as a target in the treatment of myocardial fibrosis, further exploration of the mechanisms of YAP in myocardial fibrosis may contribute to more potential therapeutic strategies for the treatment of this disease.

A study reported that TGF‐β/hypoxia‐induced fibroblast proliferation is inhibited with the downregulation of YAP, suggesting that YAP is an important molecule in promoting fibroblast proliferation.^218^ YAP has been found to be an important downstream molecule of AT1R, which can sense ECM deposition. Therefore, as ECM deposition increases, YAP can be activated and further promotes the transdifferentiation of cardiac fibroblast into myofibroblast, accelerating the development of myocardial fibrosis. And downregulation of YAP expression attenuates a series of profibrotic responses in cardiac fibroblast reactions.^219^ Indeed, the mechanism of YAP in myocardial fibrosis has been extensively investigated. In myocardial fibrosis, YAP can act as a downstream signal of Wnt and TGF‐β and contributes to the secretion of the profibrotic cytokine IL‐33 in cardiac fibroblasts, thereby promoting myocardial fibrosis.^220^ In addition, YAP also plays a significant role in activating myofibroblast and increases profibrotic genes expression through upregulating the expression of myocardin‐related transcription factor A gene.^221^ Notably, in hyperglycaemic conditions, YAP can induce a more pronounced myocardial fibrotic response. According to what was presented above, O‐GlcNAc promotes the upregulation of YAP expression. And the activated YAP further phosphorylates protein kinase B (AKT) and reduces glycogen synthase kinase‐3 β （GSK3β） activity, ultimately causing overexpression of forkhead box M1 (FOXM1) in cardiomyocytes and leading to myocardial fibrosis.^133^ These studies suggest that the absence or inactivation of YAP in fibroblasts can effectively mitigate the development of myocardial fibrosis.

Currently, drugs have been developed to target YAP for the treatment of myocardial fibrosis. It had been found that mevalonate pathway has the ability to activate YAP. And statins, the inhibitors of 3‐hydroxy‐3‐methylglutaryl coenzyme A reductase (HMG‐CoA reductase) in the mevalonate pathway, may further inhibit the upregulation of YAP expression. Lovastatin had been demonstrated to decrease YAP‐induced profibrotic genes expression and can serve as a promising candidate for myocardial fibrosis.^222^ In addition, a reduced graphene oxide/silk fibroin‐modified nanofibrous cardiac patch had been found to significantly improve myocardial fibrosis and recover cardiac function in a rat model. The expression of the YAP/TAZ–TGF‐β1/Smads signaling pathway was significantly attenuated in the improved rat.^223^

### 6.2 Pulmonary fibrosis

Idiopathic pulmonary fibrosis is a chronic progressive interstitial lung disease that is intimately related to fibroblast activation and alveolar epithelial cell senescence.^224^ Fibroblast activation causes increased collagen synthesis and ECM deposition, which can result in respiratory failure and even death. Actually, the prevalence of idiopathic pulmonary fibrosis increases with age.^225^, ^226^, ^227^ Currently, there is no effective treatment for idiopathic pulmonary fibrosis, and the available drugs can only alleviate the fibrotic process but not fully restore lung function.^228^ Patients frequently choose lung transplants to increase their longevity; nevertheless, the low rate of lung transplantation is a result of the restricted number of lung donors available, and the immune reaction following the transplant may result in a low success rate.^229^ Therefore, there is an urgent need to explore the pathogenesis of pulmonary fibrosis in order to identify effective therapeutic targets.

Presently, several studies have reported the significant role of YAP in pulmonary fibrosis, with ongoing research focusing on unraveling the mechanisms by which YAP contributes to the development of this condition. Moreover, researchers are exploring drugs that directly target the YAP or its signaling pathways as potential treatments for pulmonary fibrosis. In one study, elevated YAP activity was observed in patients with idiopathic pulmonary fibrosis, which correlated with phosphorylation of S6 and PI3K in human bronchial epithelial cells. Notably, the mTOR/PI3K/AKT signaling pathway also exhibited increased levels of S6 phosphorylation. The use of S6 phosphorylation inhibitors can decrease YAP nuclear translocation and reduce the expression of YAP targeting genes. These phenomena suggest that there is crosstalk between YAP and the mTOR/S6 signaling pathway, which can exacerbate pulmonary fibrosis.^230^ Furthermore, by downregulating YAP gene expression in bleomycin‐induced pulmonary fibrosis mice, a reduction in alveolar epithelial cell senescence and a reduction in clinical signs of pulmonary fibrosis mice can be observed.^231^ In fact, YAP is involved in pulmonary fibrosis through a variety of signaling pathways. mi‐RNA, kinases, hormone receptors had also been identified as upstream regulatory signals of YAP in pulmonary fibrosis. The Twist structural domain contains transcription factors that are involved in cell proliferation and differentiation through promoting transcriptional activation of downstream targeting genes.^232^ YAP had been found to participate in mediating the transcriptional activation of Twist through nuclear translocation and binding to TEAD to promote fibrosis. By knocking down miR‐15a, it can be observed that YAP‐mediated transcriptional activation of Twist was inhibited, thereby reducing fibrosis.^233^ In contrast to miR‐15a, the knockdown of miR‐138 activates YAP and thus promotes fibrosis. Pulmonary fibrosis‐associated RNA is a long‐stranded noncoding RNA that can act as a competing endogenous RNA for miR‐138. Therefore, upregulation of pulmonary fibrosis‐associated RNA expression can suppress miR‐138 expression, which in turn activates YAP‐induced Twist transcriptional regulation and promotes the development of fibrosis.^234^ The mechanism of miR‐107 in pulmonary fibrosis is similar to that of miR‐138, but a more specific regulatory signaling pathway had been reported. It was found that miR‐107 reduces the fibrotic phenotype of perivascular cells by inhibiting the HIF‐1α/notch homolog 1 (Notch1)/Beta platelet derived growth factor receptor (PDGFRβ)/YAP1/Twist1 signaling pathway and then inhibits the conversion of perivascular cells to myofibroblasts.^235^ Thus, different mi‐RNAs have diverse roles in YAP‐induced fibrogenesis, and potential therapeutic targets and treatments for pulmonary fibrosis need to be defined based on specific mechanisms. Aurora kinase A (AURKA) was found to reduce YAP phosphorylation and promote the expression of profibrogenic genes in lung fibroblasts. MK‐5108, an inhibitor of AURKA, was demonstrated to inhibit actin polymerization and TGF‐β signaling, thereby impairing YAP nucleus translocation and preventing it from functioning. A reduction in collagen deposition in mice was also observed through using MK‐5108, suggesting that MK‐5108 may be a hopeful drug to improve YAP‐mediated pulmonary fibrosis. In addition to AURKA, tank‐binding protein kinase‐1 (TBK1) also plays a role in YAP/TAZ‐mediated pulmonary fibrosis, as TBK1 can stabilize YAP/TAZ protein and further promote fibroblast activation.^236^ Interestingly, the regulatory roles of both AURKA and TBK1 are independent of the Hippo pathway. Furthermore, sphingosine kinase 1 (SPHK1) and its product S1P have been demonstrated to activate YAP and mitochondrial reactive oxygen species mediated by TGF‐β, which can activate fibroblasts and lead to fibrosis.^237^ Unlike AURKA and TBK1, the activation of SPHK1 is dependent on the Hippo signaling pathway. All of the above kinases play a facilitative role in pulmonary fibrosis, and therefore inhibitors targeting these kinases may be potential candidates for pulmonary fibrosis therapy. Adrenergic receptors belong to GPCRs and activation of the GPCR can cause YAP/TAZ to be phosphorylated and inhibited. Through using the adrenergic receptor agonist ACT‐333679, it can be observed that YAP/TAZ loses the ability to enter into the nucleus due to be phosphorylated, and cAMP serves as the mediator leading to nuclear exclusion of YAP/TAZ and its action is dependent on LATS in this process. The phosphorylated YAP/TAZ is unable to bind to Smad and inhibits the activations of profibrotic genes.^238^ Thus, adrenoceptor agonists may become important agents in the treatment of pulmonary fibrosis. In addition to adrenergic receptors, agonism of dopamine receptor D1 had also been shown to reverse pulmonary fibrosis by inhibiting the function of YAP/TAZ in cells.^239^

Additional drugs have been explored to alleviate pulmonary fibrosis in response to YAP/TAZ and, interestingly, herbal medicine is also thought to play an important role in alleviating YAP/TAZ‐mediated pulmonary fibrosis. Statins have been demonstrated to alleviate myocardial fibrosis and indeed, statins have also been found to play a role in improving pulmonary fibrosis. By administering simvastatin to mice with bleomycin‐induced pulmonary fibrosis, the amount of collagen deposition in the mice lungs was reduced. At the same time, YAP proteasomal degradation can be observed both in mouse fibroblast and in human fibroblast nuclei, and this degradation mediated by simvastatin was independent of the Hippo pathway while directly targeted to the mevalonate pathway.^240^ Melatonin has been found to promote the downregulation of fibrosis markers. The melatonin receptors MT1 and MT2 are also GPCRs.^241^, ^242^ Unlike statins, melatonin binding to the receptors can stimulate the activation of the Hippo pathway by the GPCR signaling pathway, thereby inhibiting YAP nuclear translocation and reducing TGF‐β‐mediated profibrosis.^243^ Icariin is a herbal medicine extracted from the Herba Epimedii.^244^ Similar to melatonin, Icariin can restrict YAP nuclear translocation by activating the Hippo pathway to reduce pulmonary fibrosis.^245^ In particular, another herb, saffron yellow, can improve pulmonary fibrosis caused by paraquat.^246^ Paraquat is a highly toxic pesticide that patient is difficult to survive once it is accidentally administered.^247^ In paraquat‐intoxicated mice, the Hippo pathway is inhibited so the YAP can locate in cell nucleus and has a crosstalk with the TGF‐β/Smad pathway and finally causes pulmonary fibrosis. Actually, saffron yellow can restore the Hippo pathway, thereby alleviating pulmonary fibrosis. However, studies have shown that the activation of YAP is regulated by multiple signaling pathways, so saffron yellow only partially reduces pulmonary fibrosis but does not completely eliminate it.^248^

### 6.3 Liver fibrosis

The pathophysiology of liver fibrosis, which is typically brought on by chronic liver damage, includes heavy alcohol use, viral hepatitis, and nonalcoholic fatty liver disease.^249^, ^250^ Prolonged liver fibrosis may lead to scarring, destruction of liver structure, complicating portal hypertension and hepatic insufficiency, and then may result in diverse tissues and organs damage, such as splenomegaly, gastrointestinal stasis, ascites, bile pigmentation, and estrogen increased.^251^ More critically, chronic liver fibrosis is exceedingly hazardous to human health since it can lead to cirrhosis and liver cancer.^252^ However, the effective antifibrotic drugs are limited. It is generally accepted that hepatic stellate cell (HSC) activation is an important factor in the development of liver fibrosis. HSC mainly acts as a store of vitamin A in normal liver while it can be activated and transdifferentiation into myofibroblasts in damaged liver. Myofibroblasts can lead to the deposition of ECM proteins and α‐SMA, ultimately resulting in fibrosis.^253^, ^254^ In addition, TGF‐β is also considered to be involved in the development of liver fibrosis by promoting the transdifferentiation of HSCs into myofibroblasts, increasing ECM synthesis, and inhibiting ECM degradation. Importantly, TGF‐β can cause fibrosis in part by acting with YAP/TAZ.^255^, ^256^

YAP/TAZ has been found to have important effects on the biological behavior of HSCs, including proliferation and differentiation. Blocking the way of YAP into the nucleus using tetramethylpyrazine can reduce the inhibitory effect of YAP on p53, whose activation can further induce HSCs senescence, thereby alleviating liver fibrosis.^257^ In addition, it was discovered that in activated HSCs, the expression of YAP became increase and inhibiting YAP expression may contribute to increasing quiescent HSCs, reducing their activation and proliferation.^258^ Dai et al.^259^ found that continuous consumption of foods in high fat content stimulated the development of insulin‐resistant liver fibrosis. By downregulating YAP expression in this insulin resistance mice model, liver fibrosis was significantly improved.^259^ In fact, YAP was found to have the opposite effect in liver fibrosis mediated by hepatic ischemia–reperfusion injury. In a mouse model with hepatic ischemia–reperfusion injury, the use of YAP inhibitors can cause hepatocyte degeneration and necrosis, and hepatic lobule congestion and edema. In contrast, the activation of YAP leaded to activation of HSCs, α‐SMA synthesis decrease, and improve liver fibrosis mediated by hepatic ischemia–reperfusion injury.^260^ Thus, YAP appears to have different stimulatory effects in diverse types of liver fibrosis, and clarification of its specific mechanism is required to explore its potential application in the treatment of liver fibrosis.

The mechanism of YAP/TAZ in liver fibrosis had been found to be related to enzymes, amino acids, and fatty acids. The expression of NUAK family kinase 1 (NUAK1) increased in fibrosis, which can be regulated by TGF‐β.^261^ The increased NUAK1 can activate the YAP/TAZ and TGF‐β/Smad signaling pathways to promote fibrosis development, and the activated YAP/TAZ can in turn act on NUAK1 to further upregulate NUAK1 expression. This positive feedback pathway exacerbates liver fibrosis and blocking NUAK1 expression significantly improves liver fibrosis.^262^ In addition, acid ceramidase expression is found to be upregulated in patients with advanced fibrosis. Using tricyclic antidepressants to inhibit acidic ceramidase in HSCs of mice with liver fibrosis results in a significant downregulation of fibrosis, stromal stiffness, and YAP/TAZ activity.^263^ Besides, Chowdhury et al.^264^ demonstrated that the NAD‐dependent deacetylase sirtuin 6 is involved in the development of liver fibrosis. Sirtuin 6 contributes to YAP/TAZ deacetylation and reprograming the TEA domain transcription factor complex composition, thereby reducing the expression of downstream liver fibrosis targeting genes and ultimately delaying the development of liver fibrosis.^264^ Furthermore, through using N‐acetyl‐l‐tryptophan in mice with CCl_4_‐induced liver fibrosis, the TGF‐ β/Smad and Hippo/YAP signaling pathways were inhibited, and consequently, the expressions of α‐SMA and collagen I proteins were reduced, contributing to liver fibrosis improvement.^265^ Indeed, fatty acids had also been found to participate in the activation of HSCs in liver fibrosis. Exogenous fatty acids can enter the cells and become lipid droplets and activate MAPK13. Activated MAPK13 promotes YAP nuclear translocation and binds to TEAD to upregulate the expression of liver fibrosis signals.^266^

A variety of antiliver fibrosis drugs targeting YAP/TAZ have been identified, including natural herbal medicines and western medicines. Many studies have demonstrated that herbal medicines play an important role in improving liver fibrosis. The Yiqi Huxue (YQHX) formulation has been found to be effective in downregulating YAP/TAZ activity and fibrosis symptoms in mice with CCl_4_‐induced liver fibrosis. Besides, in a controlled experiment in patients with liver fibrosis caused by chronic hepatitis B, fibrosis can be improved more significantly in the group taking YQHX than in the control group, and the activity of YAP in liver and plasma was inhibited through using YQHX.^267^ Besides, dihydrotanshinone I (DHI) is also an important ingredient in traditional Chinese medicine. It was proven that through the administration of DHI to rats with bile duct ligation‐induced liver fibrosis for 14 days, YAP lost the ability to bind to TEAD in the cell nucleus so the mRNA expression of its downstream profibrotic gene, such as TGFβ1, α‐SMA and COL1A1 and collagen deposition all decreased.^268^ In addition, resveratrol is considered to have hepatoprotective, antibacterial, and anti‐inflammatory effects.^269^, ^270^ In one study, resveratrol was demonstrated to activate the Hippo pathway to inhibit the YAP/TAZ nuclear translocation and promote HSCs apoptosis, contributing to decreasing in the release of collagen and α‐SMA. In fact, there is a bidirectional interaction between YAP/TAZ and resveratrol that YAP/TAZ also has an effect on resveratrol. Actually, the overexpression of YAP/TAZ can reduce the inhibitory effect of resveratrol on HSCs.^271^ A controlled assay showed that the antifibrotic mechanism of liquiritigenin is similar to that of resveratrol. Liquiritigenin can activate LATS1, and then increased the cytoplasmic content of phosphorylated YAP/TAZ. The increased YAP/TAZ in cytoplasm prevented the translocation of the Smad complex to the nucleus, thereby blocking the TGF‐β/Smad signaling pathway.^272^ Morin is a natural flavonoid that had been shown to play a role in tumor. Similarly, Perumal et al.^273^ found that morin can restrict the YAP/TAZ nuclear translocation through activating the Mst in Hippo signaling pathway and then influenced Smad complex as liquiritigenin. 3‐tigloyl‐khasenegasin F (TKF) is a natural derivative of citrulline, which can enter into hepatocytes to inhibit YAP activation and reduce the synthesis of α‐SMA and collagen. The blocked YAP signaling prevents the activation of the NICD, which can contribute to liver fibrosis‐related genes in downstream of the Notch signaling pathway silencing. Ultimately, TKF reduces HSC activation and liver injury by inhibiting both YAP and Notch signaling pathway.^274^ In addition to natural herbal medicines, many western medicines also have been found to be effective in alleviating liver fibrosis. Magnesium isoglycyrrhizate (MgIG) is a novel antifibrotic drug derived from herbal ingredients. Its mechanism is similar to most traditional Chinese herbal medicines that depend on Hippo signaling pathway. MgIG is able to promote the decrease of HSC activation factors and increase the expression of anti‐inflammatory factors by inhibiting the nuclear translocation of YAP, thus exerting both antifibrotic and anti‐inflammatory effects.^275^ In the CCl_4_‐induced liver fibrosis model, mitochondria was significantly damaged while the release of reactive oxygen species was significantly increased. By intraperitoneally injecting mitoquinone mesylate (mitoQ), mitochondrial damage and reactive oxygen species release were reduced. At the same time, the JNK/YAP signaling pathway was inhibited, which can reduce the expression of TGF‐β and the accumulation of collagen, suggesting that mitoQ may be a potential effective antifibrotic agent.^276^ In addition, Lu et al.^277^ found that the proton pump inhibitor pantoprazole also showed antifibrotic effect through downregulating YAP. The decreased YAP inhibited the expression of downstream profibrotic genes. Interestingly, Chlorella extract, a beneficial food, has been proven that has an antifibrotic mechanism, which is similar to MgIG, implying that in the future it may be possible to improve fibrosis by consuming appropriate foods.^278^, ^279^ Specifically, chlorella extract can also reduce the release of reactive oxygen through the FOXO1/p‐AMPK axis.^279^

### 6.4 Renal fibrosis

Renal fibrosis is an important pathological feature of many chronic kidney diseases, end‐stage renal disease, and diabetic nephropathy.^280^, ^281^, ^282^ The manifestations of renal fibrosis include glomerulosclerosis, tubular atrophy, and interstitial fibrosis.^283^ Prolonged renal fibrosis greatly damages the regenerative potential of the kidney and disrupts normal kidney function.^284^ At present, dialysis and kidney transplantation are the most important methods to treat renal fibrosis.^285^ However, patients are severely constrained from receiving treatment due to the high cost, a lack of medical resources, and kidney supplies. Investigating effective antifibrotic medicines is necessary now. There is growing evidence that the development of renal fibrosis is closely related to ECM deposition, epithelial–mesenchymal transition of renal tubules, and fibroblast‐myofibroblast transdifferentiation.^286^, ^287^, ^288^, ^289^ Identifying the specific mechanisms underlying the development of renal fibrosis may provide more reliable targets for antifibrotic therapy.

The profibrotic effect of YAP/TAZ in the kidney had been discovered by numerous investigations. It was discovered that TGF‐dependent renal fibrosis results in an upregulation of TAZ. Activated TAZ can encourage the secretion of its downstream profibrotic proteins, such as CTGF, fibronectin, and plasminogen activator inhibitor‐1.^290^ Chen et al.^291^ demonstrated that specific deletion of YAP in diabetic mice significantly ameliorated the extent of interstitial fibrosis, and similarly, α‐SMA, CTGF, and collagen expression were subsequently downregulated, implying that the transdifferentiation of fibroblast into myofibroblast was inhibited. Also, through using the YAP inhibitor VP, a similar phenomenon can be observed.^291^ In addition, in mice with unilateral ureteral obstruction (UUO)‐induced renal fibrosis, YAP/TAZ deficiency can also reduce endothelial–mesenchymal transition, ECM deposition, and fibroblast‐myofibroblast transdifferentiation and finally improve renal interstitial fibrosis.^292^, ^293^

The profibrotic effects of YAP/TAZ in the kidney are closely linked to multiple signaling pathways. Exploring the upstream/downstream molecules of YAP/TAZ may provide new ideas for antifibrotic therapies. A study found that YAP/TAZ participates in the response of TGF‐β to mechanical stimuli. With matrix stiffness enhancing, TGF‐β can activate Smad2/3 in a phosphorylated manner and the activated Smad2/3 subsequently translocates to the nucleus. YAP/TAZ is able to maintain the nuclear accumulation of Smad2/3 and promotes the TGF‐ β/Smad signaling pathway, leading to the development of renal fibrosis.^294^ Recent studies had shown that YAP/TAZ and TGF‐β can also mediate renal fibrosis in a non‐Smad‐dependent manner. In renal mesenchymal fibroblasts NRK‐49F, TGF‐β can interact with p38 MAPK to activate YAP and promotes the nuclear translocation of YAP. Activated YAP further upregulates transient receptor potential channel 6 (TRPC6) expression, which ultimately drives fibroblast transdifferentiation, manifesting as α‐SMA expression and collagen I secretion.^295^ In addition, TGF‐β‐induced YAP/YAZ activation in NRK‐49F cells can also be regulated by the Rictor/mTORC2/Akt signaling pathway. This mTORC2 signaling pathway is activated by TGF‐β and then upregulates YAP/TAZ expression and CTGF secretion.^296^ In particular, Wnt signaling pathway can also participate in the interaction between TGF‐β and YAP/TAZ.^297^ Piezo1 is a receptor on the cell membrane, which can respond to mechanical stimulation.^298^ In response to the increased matrix stiffness, piezo1 also accelerates the progression of renal fibrosis via p38 MAPK/YAP like TGF‐β.^299^ Moreover, inhibiting the Hippo signaling pathway is key for YAP/TAZ to exert its profibrotic effects in the kidney, and there are also many upstream molecules that act on the Hippo signaling pathway to affect YAP/TAZ activity.^300^ In a mouse model of ischemia–reperfusion‐induced renal interstitial fibrosis, the expression of the transcription factor KLF4 is upregulated and then increases ITCH expression. In the function of itchy E3 ubiquitin protein ligase (ITCH), LATS1 is degraded so it is unable to phosphorylate and inhibit YAP/TAZ. KLF4 is also able to directly bind to the promoter of YAP to increase YAP expression. And in the action of both these two pathways, YAP/TAZ is strongly activated and exacerbates renal fibrosis.^301^ In addition, Kindlin‐2 was found to promote the combination of MOB‐1 and the E3 ligase praja2 to promote MOB‐1 degradation, leading to a decrease in LATS phosphorylation and an increase in YAP nuclear translocation. At the same time, the downregulation of Kindlin‐2 can activate the Hippo signaling pathway and reduce renal fibrosis.^302^ In hypertensive nephropathy, the increase of angiotensin II levels is significant. Angiotensin II is able to upregulate the expression of profibrotic genes by inhibiting the phosphorylation of LATS and YAP and promoting the translocation of YAP to the nucleus and its binding to TEAD.^303^ Furthermore, Rac‐GTPase, a member of the Rho‐GTPase family, was shown to activate YAP/TAZ in a model of renal fibrosis, and at the same time, Rac‐GTPase also mediated the production of free radicals that contributes to the phosphorylation and activation of EGF receptor (EGFR).^304^ And the mechanism is described in more detail in a study by another group. RhoA is the most abundant Rho‐GTPase in the renal proximal tubule.^305^ Chen et al.^291^, ^306^ found that activation of both the EGFR/PI3K/Akt signaling pathway and the RhoA/Rock signaling pathway in diabetic renal proximal tubular epithelial cells promoted YAP activation and there was a crosstalk between these two signaling pathways. In addition to these signaling pathways, both FGF2/STAT3 and YTH domain‐containing protein family (YTHDF1) can also exacerbate renal fibrosis by upregulating YAP expression.^307^, ^308^ In conclusion, there are many YAP/TAZ signaling pathways involved in renal fibrosis, so exploring drugs that inhibit the activation of profibrotic YAP/TAZ signaling pathways may open the way for antifibrotic therapy.

As research has progressed, a number of potential drugs and pathways have been identified that act on alleviating renal fibrosis by downregulating YAP/TAZ. VP is the most typical inhibitor of YAP. Using VP in mice with UUO‐induced renal fibrosis can observe a significant improvement in renal fibrosis extent. VP can inhibit YAP interaction with TEADs so YAP fails to mediate the TGF‐β/Smad pathway, resulting in fibroblast activation decrease and ECM deposition.^309^ Polydatin (PD) is a natural active ingredient in the thuja family of plants. A study demonstrated that the mechanism of PD inhibiting YAP activity and nuclear translocation is similar to VP, but its function in reducing α‐SMA expression and collagen I deposition was more pronounced compared with VP.^310^ In the activated Hippo signaling pathway 14‐3‐3ζ can bind to YAP/TAZ in the cytoplasm and contributes to YAP/TAZ degradation. It was found that when in a mouse model of acute kidney injury with 14‐3‐3ζ overexpression, the expression of fibrosis‐related proteins was downregulated and interstitial fibrosis was alleviated.^311^ The farnesoid X receptor (FXR), a nuclear receptor, is a multifunctional transcription factor that has been found to have an important role in antifibrosis.^312^ The use of FXR agonists in UUO‐induced interstitial fibrosis mice can reduce fibrosis in a dose‐dependent manner.^313^ Further studies found that FXR agonists reduced the activity of the nonreceptor tyrosine kinase Src and prevented the nuclear translocation of YAP by phosphorylating YAP at Ser 127, ultimately blocking the progression of renal fibrosis.^314^ Notably, exosomes can mediate the transfer of macromolecules between cells, such as nucleic acids and enzymes. Antifibrotic therapy using exosomes has become an emerging therapeutic modality.^315^ Studies have demonstrated that through using exosomes to transfer miR‐374a‐5p from mesenchymal stem cells to human renal tubular epithelial cell line (HK‐2), miR‐374a‐5p can reduce the apoptosis of HK‐2 by binding to MAPK mRNA and regulating the MAPK6/MK5/YAP axis, and finally alleviates renal fibrosis.^316^ In addition to miR‐374a‐5p, exosomes constructed from human umbilical cord MSCs can also deliver CK1δ and E3 ubiquitin ligase β‐TRCP. Not only have human umbilical cord MSC exosomes been shown to limit nuclear translocation of YAP, but CK1δ/β‐TRCP can also promote ubiquitination and degradation of YAP in the cytoplasm. Therefore, the synergistic effect of exosomes and CK1δ/β‐TRCP contributes to significant improvement of renal fibrosis in a UUO mouse model.^317^

**FIGURE 6:**
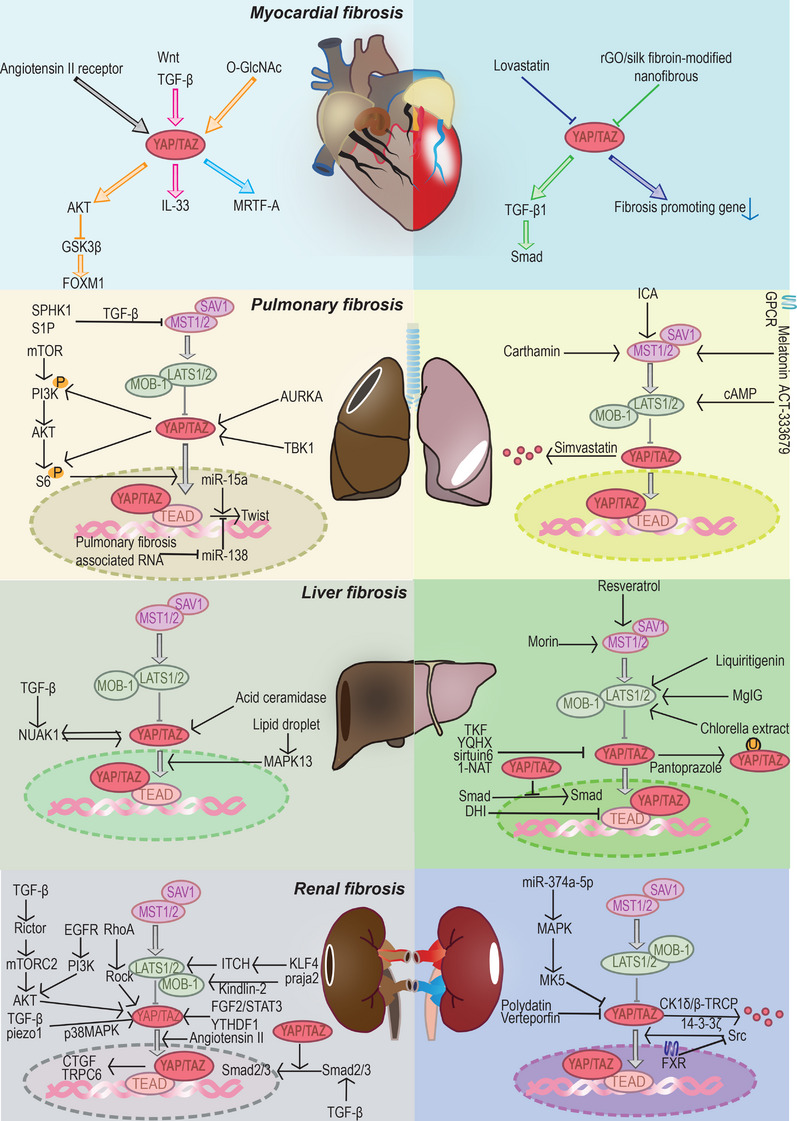
The role of YAP/TAZ in fibrosis development and antifibrosis drugs targeting YAP/TAZ and Hippo signaling pathway. Many upstream signals can activate YAP/TAZ directly or inhibit Hippo signaling pathway to stimulate the transdifferentiation of fibroblast into myofibroblast and cause ECM deposition, finally resulting in aggravation of fibrosis. Notably, not only western drugs but also traditional Chinese medicines are widely used in antifibrosis therapy, which can inhibit the activation of YAP/TAZ or promote YAP/TAZ degradation.

In short, numerous effective medications targeting YAP/TAZ or its signaling pathway have been discovered, and the mechanism of YAP/TAZ in the development of fibrosis has been extensively explored (Figure 6). To study the effects of pesticides, many medications are now exclusively tested on mice, and the number of medications that enter clinical trials is restricted. Therefore, more research is needed to determine the safety and dependability of these medications.

## 7 YAP/TAZ AND ORGAN REGENERATION

There is a double effect of YAP/TAZ on cell biological behavior. Excessive cell proliferation and abnormal differentiation can cause cancer and fibrosis, while the normal proliferation and differentiation of cells in aging or damaged organs are conducive to organ regeneration.

### 7.1 Heart regeneration

Myocardial infarction may lead to massive myocardial cell, fibroblast, and endothelial cell death.^318^ Myocardial cells actually have a restricted capacity for regeneration because they are highly specialized cells. More than 99% myocardial cells can only increase in size but lack the ability to differentiate and proliferate.^319^, ^320^ As a result, after myocardial damage, the heart is usually repaired by collagen and is unable to regenerate through surrounding homogeneous cells. Eventually, the heart may become fibrotic and hypertrophic, causing heart failure.^321^ Heart transplantation and aided device therapy to the left ventricle are two invasive operations that are nearly always necessary for patients with heart failure to sustain cardiac function.^322^ A growing number of studies have demonstrated that mammalian neonatal cardiomyocytes still have the potential to proliferate in the first week of life, and this capacity disappears after one week.^323^, ^324^, ^325^, ^326^, ^327^ Meanwhile, newly generated myocardial cells are thought to be derived from preexisting myocardial cells.^328^ Therefore, promoting myocardial cell proliferation to induce the adult heart regeneration may be an extremely promising approach for the future treatment of myocardial infarction and heart failure.^318^, ^329^ Through constructing YAP overexpressing transgenic mice YAP5SA, a study has found that the myocardial cells were reprogrammed into fetal‐like cells and the myocardial cell could enter the cell cycle multiple times to proliferate significantly. In addition, the myocardial cells that have proliferated were able to couple to the existing cardiomyocytes and contract.^330^ This finding revealed a significant role for YAP in mediating myocardial cell proliferation. Lin et al.^331^ showed that activation of YAP in a mouse model of myocardial infarction can limit the infarct size and improve cardiac function, and the activated YAP promotes myocardial cell proliferation primarily rather than inhibiting apoptosis. Traditionally, it is considered that YAP contributes to stimulating myocardial cell proliferation but has no effect on hypertrophy and the role of YAP in myocardial cell proliferation is closely related to TEAD.^331^, ^332^ However, recent studies have proven that YAP is also activated in pathological cardiac hypertrophy. Additionally, extending the period that YAP is activated causes mitochondrial damage and pathological cardiac hypertrophy. Furthermore, the mitochondrial damage also inhibits the role of YAP in mitosis.^333^ Therefore, it is crucial to understand the mechanism of YAP in cardiac regeneration and to precisely control the time of YAP activation to induce myocardial cell proliferation rather than hypertrophy in cardiac regeneration.

Small noncoding RNAs (miRNAs) are closely linked to the heart regeneration actions of YAP. A growing number of studies have proven that miRNAs play an important role in cardiovascular disease. In particular, miRNAs have been found to promote myocardial cell proliferation.^334^, ^335^, ^336^ Different miRNAs can influence YAP through different mechanisms. miR‐199a‐3p directly inhibits TAOK1‐3 and STK38L, which are phosphorylation‐promoting molecule upstreams of YAP, and the E3 ubiquitin ligase β‐TrCP, which can mediate YAP degradation, thereby promoting YAP nuclear translocation and myocardial cell proliferation.^337^ In addition, miR‐302d, miR‐373, and miR‐590‐3p can not only inhibit STK38L but also directly inhibit LATS in the Hippo signaling pathway, thus facilitating YAP activation. In fact, it was shown that the activation of YAP is necessary for the above miRNAs to induce myocardial cell proliferation.^337^ Nugroho et al.^338^ found that overexpression of miR‐411 in the heart significantly improved the heart phenotype after myocardial infarction and in this process, miR‐411 directly regulated Foxo1 expression and then enhanced YAP activity and nuclear translocation, ultimately promoting myocardial cell proliferation and inhibiting myocardial cell apoptosis. At present, upstream signals that can regulate the interaction of miRNA and YAP has been found. Toll‐like receptors (TLRs) have been found to be involved in the metabolic reprogramming.^339^ Activating TLRs with the TLR ligand Poly(I: C) promotes cardiac regeneration. And Poly(I: C) can reverse LATS phosphorylation in a glucose‐dependent manner, thereby promoting YAP nucleus translocation. In the nucleus, YAP binds to TEAD and activates miR‐152, which inhibits the cell cycle inhibitory molecules P27klp1 and DNMT1 outside the nucleus, thereby promoting myocardial cell entry into the cell cycle and proliferation.^340^ In addition to TLR, melatonin also binds to its receptors M1 and M2 and the activating effect of melatonin on YAP is achieved by inhibiting the activity of miR‐143, which ultimately accelerates myocardial cell proliferation.^341^ Besides, methyltransferase‐like 3 (METTL3) can also regulate miR‐143. Deletion of METTL3 promotes the synthesis of primary miR‐143 (pri‐miR‐143) and inhibits its maturation into miR‐143. Due to the decreased miR‐143, YAP can be activated and then promotes myocardial cell proliferation.^342^ Thus, YAP and miRNA signaling pathways may be potential targets for cardiac regenerative therapies. In addition to miRNAs, lncRNAs also have a regulatory role in the promotion of cardiac regeneration by YAP. During cardiac development, lncRNA LncDACH1 is upregulated and diminishes cardiac regenerative potential, whereas conditional knockdown of LncDACH1 in the heart restores cardiac regenerative capacity.^343^ Furthermore, it was found that the knockdown of LncDACH1 enhanced protein phosphatase 1 catalytic subunit alpha (PP1A) activity and induced YAP dephosphorylation, thereby facilitating the activation of myocardial cell proliferative capacity.^343^

YAP‐induced myocardial cell proliferation is also tightly correlated with enzymes, certain proteins, receptors, and hormones, which can be employed as new targets for heart regeneration. It was found that GSK‐3 inhibitor GSK‐3I can promote myocardial cell proliferation mediated by β‐catenin and the proliferative potential of myocardial cell was more pronounced when YAP was activated. Therefore, blocking GSK‐3 and activating YAP can play a synergistical role.^344^ Pik3cb can encode the catalytic subunit of phosphoinositol‐3‐kinase (PI3K).^345^ Lin et al. demonstrated that pik3cb was a direct target of YAP in the regulation of myocardial cell proliferation. Activated YAP binds to TEAD in the nucleus and through directly binding to the enhancer of pik3cb, YAP/TEAD can increase the function of pik3cb. And then the activated pik3cb can induce myocardial cell proliferation and reduce cardiomyocyte apoptosis through the PI3K–Akt pathway.^346^ LKB1 is able to directly phosphorylate AMPK, and a study reported that the loss of LKB1 function is benefit in AMPK dephosphorylation and promotes myocardial cell to re‐enter the cell cycle by activating YAP. However, inhibiting YAP activity can reverse the effect of downregulated LKB1 on myocardial cell proliferation.^119^ Besides, Li et al.^347^ demonstrated that the downregulation of α‐catenin may contribute to enhancing nuclear translocation of YAP and increasing proliferation of myocardial cell. α‐Catenin‐deficient patients showed a more primitive type of myocardial cell with an increased translocation of YAP into the nucleus. In the patients with this disorder, myocardial cells achieve significantly proliferative potential.^347^ Moreover, vestigial like 4 (VGLL4) is a protein containing the Tondu structural domain, which can bind to TEAD. VGLL4 with Tondu domain acetylation was found that have the ability to inhibit YAP/TEAD interaction and affect cardiac regeneration by inducing myocardial cell apoptosis.^348^ ERBB2 is the tyrosine kinase receptor for neuromodulin‐1. In myocardial cell, ERBB2 signaling can effectively mediate myocardial cell proliferation, and ERK‐mediated YAP activation is a necessary downstream target for ERBB2 signaling.^122^ In addition, the Gaq protein‐coupled receptor P2Y_2_R also has a regulatory role in YAP. Unlike ERBB2, activation of YAP by P2Y_2_R is Hippo signaling pathway dependent. P2Y_2_R can be activated by UTP and subsequently inhibits the Hippo signaling pathway by downregulating MST1 and LATS1 to induce nuclear translocation of YAP, thereby promoting the proliferation and migration of human cardiac progenitor cells in favor of facilitating cardiac regeneration.^349^, ^350^ Furthermore, using metoprolol to inhibit adrenergic receptors was also found to enhance myocardial cell regeneration ability in mice through the β1‐AR–Gαs–YAP signaling axis.^351^ In addition to these molecules, hormones are also an important class of regulators. Gan et al.^352^ demonstrated that glucocorticoids play a negative regulatory role in myocardial cell proliferation. Glucocorticoids are able to induce the cAMP/PKA‐mediated phosphorylation of YAP, thereby inhibiting the expression of the targeting genes in the downstream.^352^ In contrast to the effect of glucocorticoids, progesterone can upregulate YAP activity and increase the proliferative potential of myocardial cell.^353^ In conclusion, YAP is a key factor in cardiac regeneration and is regulated by a variety of signals. By targeting the signaling pathways and increasing the activity of YAP, the proliferative potential of myocardial cell can be highly stimulated, thus contributing to the repair of the heart and enhancing cardiac function.

### 7.2 Intestinal regeneration

Ulcerative colitis is a group of inflammatory bowel diseases. Ulcerative colitis is prone to progress to colon cancer and an important feature of ulcerative colitis is impaired epithelial regeneration.^354^, ^355^ Currently, accelerating intestinal epithelial repair to promote mucosal healing is an important treatment for ulcerative colitis.^356^ The intestinal epithelium is one of the most self‐renewing types of tissue in mammals. YAP/TAZ had been found to play an important role in regulating intestinal regeneration and cancer. YAP has been reported that has the ability to promote crypt regeneration by inhibiting Wnt signaling and reducing the differentiation of Lgr5+ intestinal stem cells (ISCs) into Paneth cells. At the same time, intestinal regeneration is closely associated with Egfr signaling activated by YAP.^357^ In addition, YAP/TAZ has a reprogramming effect on ISCs through transforming them into a less differentiated state. During the repair process of colitis tissue, YAP/TAZ is activated to reprogramme ISCs to achieve a higher proliferative potential and promote colonic regeneration.^358^ Thus, YAP/TAZ promotes intestinal regeneration by reprogramming ISCs on the one hand and by reducing the differentiation of ISCs into Paneth cells on the other. During intestinal regeneration, downregulation of YAP/TAZ may lead to abnormal Paneth cell differentiation and ISCs reduction, which ultimately affects the regenerative effect.^359^

Indeed, the regulatory role of YAP/TAZ in intestinal regeneration is also closely linked to multiple signals. In particular, the Wnt/β‐catenin signaling pathway plays an important regulatory role in the intestinal regeneration activated by YAP/TAZ. Inhibition of YAP/TAZ was found that may cause crypt stem cells apoptosis and abnormal development of the lateral folds of the ascending colon, implying the importance of YAP/TAZ in mediating regeneration in areas of tissue damage. Actually, the expression levels of YAP/TEAD could be regulated by Wnt signaling to enhance the proliferative potential of crypt stem cells. Furthermore, in the damaged intestinal tissues, Src signaling can mediate YAP nuclear translocation, and the mediation of Src signaling is required to stimulate crypt stem cell proliferation.^28^ In addition, YAP also has a regulatory role in Wnt/β‐catenin signaling during intestinal regeneration. Deng et al.^360^ reported that YAP was downregulated in more than 60% of ulcerative colitis specimens and its reduced expression was associated with epithelial cell proliferation decrease. Through constructing YAP phosphorylation model, it can be observed that YAP is able to stimulate β‐Trcp to disrupt β‐catenin to block the activation of Wnt signaling and ultimately inhibited the proliferation of intestinal cells, implying that YAP plays a role in activating the Wnt/β‐catenin signaling pathway to affect intestinal regeneration.^360^ The study also found that YAP can stimulate the combination of β‐catenin and TCF4 to promote intestinal epithelial cell proliferation.^360^ In fact, YAP and β‐catenin can also be regulated by miRNAs. Study found that miR‐590‐3p could inhibit the activity of LATS with the involvement of various cytokines, such as TNF‐α and IL‐6, thereby stimulating the translocation of YAP and β‐catenin into the nucleus, and in the cell nucleus, β‐catenin binds to TCF and mediates intestinal regeneration.^361^ Besides, enzymes play a significant role in stabilizing and promoting YAP/TAZ‐stimulated intestinal regeneration. Protein phosphatase magnesium‐dependent 1A (PPM1A) eliminates YAP phosphorylation and promotes YAP nuclear translocation. In the PPM1A‐depleted mouse model, YAP nuclear translocation was blocked and the mouse intestinal crypts were hypoplastic, thereby causing cupped cells decrease and intestinal epithelial proliferation inhibition.^362^ Similarly, structural abnormalities in the small intestinal crypts can be observed in RhoA knockout mice, with a significant reduction in crypt cell proliferation, a decrease in the number of ISCs, and downregulations of YAP and its downstream EREG expressions. However, recovering the activation of YAP in RhoA knockout mice can reverse these phenotypes, implying that RhoA lies at the upstream of YAP to influence intestinal regenerative potential.^363^ In addition, Deng et al.^364^ showed that YAP could interact with signal transducer and activator of transcription 3 (STAT3) in the nucleus and activates STAT3 signaling to upregulate the expression of prointestinal epithelial cell proliferation genes. Interestingly, group 3 innate lymphoid cells were also proven to regulate intestinal epithelial regeneration by amplifying the effects of YAP activation in colonic crypt cells, and this pathway activated Src kinase while was independent of STAT3 signaling.^365^ Jin et al.^366^ demonstrated that prostaglandin E2 signaling facilitated the upregulation of YAP expression and increased prostaglandin‐endoperoxide synthase 2 gene (PTGS2) and prostaglandin E receptor 4 gene expression to promote colonic regeneration, whereas prostaglandin E2 signaling failed to induce colonic regeneration in YAP‐deficient cells. Moreover, bile acids can also activate Src and YAP by binding to bile acid GPCRs, thereby increasing the proliferative potential of ISCs.^367^ In fact, there are various ways to regulate YAP/TAZ in intestinal cells, ranging from directly targeting YAP/TAZ to regulate YAP/TAZ activity by inhibiting the Hippo signaling pathway, to affect YAP/TAZ by crosstalk with other signaling pathways.^368^ Even though YAP/TAZ plays such a critical role in intestinal regeneration, its continued overexpression may lead to the overproliferation of cells and the development of colon cancer. Therefore, according to the mechanism in intestinal cell proliferation, controlling the boundary between YAP/TAZ in intestinal regeneration and colon cancer is the key to the positive therapeutic effect of YAP/TAZ.

### 7.3 Liver regeneration

The treatment of many liver diseases relies on liver regeneration such as chronic liver disease, alcoholic hepatitis, liver cancer, and liver toxicity.^369^ In fact, the liver itself has highly regenerative ability and this process is complex and tightly regulated.^370^ The treatment of many liver diseases requires surgical removal of the damaged liver and then relies on the regeneration of the remaining liver in order to obtain sufficient liver function. So the potential and effectiveness of liver regeneration determine whether the patient can be treated by surgical resection and the patient's recovery and prognosis.^371^ It has been reported that YAP nuclear translocation significantly increases after hepatectomy. Although YAP/TAZ is unnecessary in liver regeneration, it plays an important role in promoting the entry of hepatocytes into the cell cycle for proliferation and in recovering the liver to normal size after hepatectomy.^372^, ^373^ In addition, further studies revealed that liver regeneration was diminished for the first 32 hours after YAP knockdown and then returned to the normal regeneration process, implying that YAP is only necessary for hepatocyte entry into the cell cycle, particularly the S phase, but not for the subsequent M phase.^374^ Although YAP/TAZ is not a necessary factor for the subsequent phases of the cell cycle, calcitonin gene‐related peptide‐receptor activity‐modifying protein 1 (RAMP1) has been shown to stimulate the proliferative process by regulating YAP/TAZ activity during subsequent phases of hepatocyte proliferation.^375^ One of the most common complications of hepatectomy is liver ischemia/reperfusion injury. Notably, HSCs proliferate significantly and YAP/TAZ is activated in ischemia/reperfusion‐injured livers. The proliferation of HSCs was reduced through administering the YAP/TAZ inhibitor VP.^376^ Thus, YAP/TAZ can regulate not only hepatocyte but also HSC proliferation for liver regeneration. Besides, hepatic cholestasis is a chronic liver disease that can lead to hepatocyte damage. A mouse model of hepatic cholestasis was constructed by knocking out the immunoglobulin kappa j region (Rbpj) to block the Notch signaling pathway. The increased bile acids in the liver can stimulate the expression of the scaffolding protein IQGAP1, leading to YAP nuclear translocation increase and ultimately increased cell proliferation in the liver.^377^ The activation of YAP in alcoholic hepatitis can also stimulate liver regeneration and promote detoxification after alcohol intoxication.^378^

Indeed, YAP/TAZ regulates liver regeneration by complex and diverse mechanisms. In contrast to YAP/TAZ, TGF‐β plays a role in inhibiting hepatocyte growth during liver regeneration.^379^ YAP/TAZ and TGF‐β also have a crosstalk during liver regeneration. In posthepatectomy hepatocytes, YAP was found to promote the translocation of the TGF‐β‐regulated transcription factor pSmad2 into the nucleus so that hepatocytes could undergo the epithelial–mesenchymal transition to break the TGF‐β‐mediated growth inhibition and eventually hepatocyte proliferation and promotes the resected liver regeneration.^380^ Peroxisome proliferator‐activated receptor α (PPARα) is considered that plays an important role in liver regeneration. In terms of specific mechanisms, PPARα is able to stimulate YAP nuclear translocation and induce the binding of YAP and TEAD, whereas knockdown of YAP can observe that periportal hepatocyte proliferation is significantly inhibited, implying that PPARα promotes liver regeneration by activating YAP.^381^ The constitutive androstane receptor has a similar role to PPARα.^382^ Notably, Ju et al.^383^ reported that Poly(ADP‐ribose) Polymerase‐1 (PARP1) is also an essential component of liver regeneration. Blocking PARP1 can reduce hepatocyte proliferation through inhibiting the expression of downstream cell cycle‐related proteins by affecting the activity of YAP.^383^ Serotonin has also been proven to regulate liver regeneration through the serotonin‐pERK‐YAP axis.^384^ Interestingly, there is also a loop pathway in the YAP/TAZ‐induced liver regeneration. It has been shown that the binding of YAP/TAZ and TEAD can directly block NR4A1 transcription, while YAP can also stimulate NR4A1 phosphorylation and nuclear translocation under the mediation of AKT, thereby inhibiting the apoptosis‐promoting effect of NR4A1 and promoting hepatocyte proliferation, and the protein tyrosine phosphatase nonreceptor type 11 inhibition amplifies the effect of YAP on NR4A1.^385^ NR4A1 can also regulate the ubiquitinated degradation of YAP through a negative feedback mechanism to prevent excessive proliferation of hepatocytes and avoid the occurrence of HCC.^386^ Notably, there are also many negative regulators in liver regeneration. Unlike PPARα, which promotes liver regeneration, the use of troglitazone, a PPARγ agonist, can inhibit hepatic elliptical cell proliferation by arresting the cell cycle in the G0/1 phase mediated by Hippo/YAP.^387^ Besides, bromodomain and extra terminal protein inhibition is also thought to affect liver regeneration by inhibiting the YAP/TAZ–Notch1–NICD axis, while YAP/TAZ overexpression can reverse the effects of BET protein inhibitors.^388^ Therefore, these drugs, which can affect liver regeneration, should be used with caution in the treatment of liver diseases to avoid compromising the therapeutic effect and even endangering life due to poor regeneration.

### 7.4 Cartilage regeneration and bone remodeling

Cartilage regeneration and bone remodeling are necessary for fracture repair. YAP/TAZ has been demonstrated to regulate cartilage regeneration through modulating chondrocyte proliferation and maturation. In addition to fracture repair, cartilage regeneration is also benefit in the treatment of osteoarthritis (OA). The process of bone remodeling contains bone absorption and new bone formation. Indeed, YAP/TAZ plays an important role in regulating the balance of osteocyte, osteoblast, osteoclast to promote bone remodeling. Particularly intriguing are the roles played by YAP/TAZ in the development and remodeling of the craniofacial and dental structures.

In fact, the role of YAP in chondrogenesis is relatively obvious while the mechanism of TAZ is still under study. Interestingly, YAP has opposite effects on chondrocytes at different stages. In detail, YAP/TEAD directly regulates Sox6 expression and promotes chondrocyte proliferation at an early stage, while YAP/Runt‐related transcription factor 2 (Runx2) reduces Col10a1 expression, thereby inhibiting chondrocyte maturation.^389^ Besides, YAP also has opposite effects on chondrocytes in different processes. The overexpression of YAP increases the proliferation of chondrocyte while suppresses the differentiation of chondrocyte through interacting with the Wnt/β‐catenin signaling pathway.^390^ Recently, TAZ has also been found to play an important role in chondrocytes. In contrast to YAP, overexpression of TAZ increases the expression of Col10a1, thereby promoting the maturation of chondrocytes. In addition to this, chondrocyte proliferation can be conspicuously accelerated by TAZ ectopic expression.^391^ The HIF‐α pathway also has an impact on the growth of chondrocyte, which can associate with YAP signaling.^14^ In chondrocytes, under mechanical stress, the expression of HIF‐α depends on the activation of YAP.^392^ Not only Hypoxia but also SETD7 expression can increase the level of HIF‐α and YAP complex, thereby contributing to chondrocyte growth and differentiation.^393^ Due to the role of YAP in cartilage, it may serve as a therapeutic target to relieve OA.

However, various investigations appear to support the opposite finding. A study showed that YAP maintains the stability of articular cartilage during OA through the antagonistic action to NF‐κB signaling, and the deficiency of YAP may expedite cartilage degradation.^394^ Nevertheless, another study considered that the partial inhibition of YAP through SiRNA combats cartilage degradation.^395^ Further research may be needed to determine the full mechanism of function of YAP in OA and to find new treatments to prevent cartilage degradation in OA.

There are three main types of cells in bone tissue, including osteocytes, osteoblasts, and osteoclasts. Only the osteocytes are located in the bone tissue while the other cells are situated on the edge of the bone tissue.^396^ And numerous studies had emphasized the significance of YAP/TAZ in these three types of cells.

Osteocytes are sensitive to mechanical signals while YAP/TAZ can act as an important component in mechanical conduction, indicating that YAP/TAZ may play a crucial role in bone remodeling mediated by osteocytes. TGF‐β acts as an inducible factor for the expression of matrix‐remodeling enzymes in osteocytes while using 8kbDMP1‐Cre to suppress the activation of YAP/TAZ in the osteocyte of mice can inhibit the expression of matrix‐remodeling enzymes, pointing out that the interaction between YAP/TAZ and TGF‐β can regulate the function of osteocytes.^397^, ^398^ Additionally, in osteocytic MLO‐Y4 cells, the shear stress‐induced activated YAP/TAZ participates in the expression of chemokines M‐CSF and Cxcl3 by mechanical induction in osteocyte‐like cells.^399^ All these evidences obviously show that the capabilities of osteocytes in increasing the osteoblasts and decreasing the osteoclast can be mediated by YAP/TAZ.

Interestingly, YAP/TAZ had diverse effects on osteoblasts at different periods. To be specific, on the one hand, YAP/TAZ induces the inhibition of Wnt signaling and Runx2 activity in osteoblast progenitors so the ability of osteoblast progenitor cells to differentiate into osteoblasts was significantly reduced.^400^ On the other hand, YAP/TAZ enhances the capacity of mature osteoblasts, contributing to bone formation.^398^ However, the role of YAP/TAZ in osteoblast differentiation is regulated by various substances. Integrin αv had been proven that has the ability to respond to mechanical stimulation. Under the mechanical stimulus, the Integrin αv can increase the activation of YAP/TAZ through Src–JNK–YAP/TAZ pathway.^401^ In addition, the osteoblast differentiation in periodontal ligament stem cells heightens the function of CTHRC1 in regulating the TAZ, and this discovery may provide a constructive approach to periodontal tissue regeneration.^402^ As the inducer of LPR6 and DVL3, YAP/TAZ contributes to osteoblast differentiation by indirectly regulating the Wnt/β‐catenin signaling pathway.^403^ More recently, the study found that the synergy between BMP‐2 pathway‐associated Smad1/5/8 heteromeric complexes and YAZ/TAZ induces the activation of osteogenic genes while Fisetin reduces the expression of osteogenic genes through suppressing the YAP.^404^, ^405^

Actually, the balance between osteoblast and osteoclast is the key to the stability of bone tissue. Now, several studies had proved that YAP is an essential factor in the formation and differentiation of osteoclasts. Knocking down the YAP in bone marrow‐derived macrophages (BMM) results in the inhibition of multinucleated osteoclast formation.^406^ Similarly, the BMM treated with VP, which prevents the binding of YAP and TEAD, can also become conscious of the reduction of osteoclast formation.^406^ Additionally, the activation of YAP has the ability to upregulate the GDF15, which had been evidenced that contributes to the osteoclast differentiation of the murine macrophage cell line RAW264.7 cells.^407^ The mechanism of YAP/TAZ on osteoclasts remains to be further investigated, and elucidation of this mechanism may provide new insights into osteoclast‐related diseases.

The role of YAP/TAZ in craniofacial and dental remodeling had been explored with further understanding of YAP/TAZ. Presently, a number of studies had identified a vital role of YAP/TAZ in craniofacial development and remodeling. YAP/TAZ participants in vasculogenesis, smooth muscle differentiation, and cerebellar development in cranial neural crest and has a significant effect on neural crest‐derived craniofacial development.^408^ Moreover, in the posterior palatal shelves, YAP/TAZ can regulate the expression of Ibsp, Phex, and Loxl4, which catalyzes collagen crosslinking, thereby elevating the palatal shelf and mineralizing the bones of the secondary palate.^409^ Furthermore, YAP/TAZ is also a participant in dental development. In ameloblasts of the mandibular first molar in rats, the staining of YAP/TAZ can be observed by hematoxylin and eosin staining, indicating that YAP/TAZ may be a significant element in the development of enamel.^410^ In fact, the expression of YAP/TAZ varies in different teeth. As for molars, both in ameloblasts and in differentiated ameloblasts the YAP plays a role while the expression of YAP is lacking in the differentiated ameloblasts in incisors.^411^ In addition to these, YAP/TAZ had been proven that has an influence on mediating cell proliferation, differentiation, and maturation in the periodontium, what is more, YAP serves as a pivotal factor in regulating the shape of the tooth crown.^412^ Under normal circumstances, the primary enamel knot (PET) will disappear between the cap and bell stages, however, the PEK remains active until the bell stage if the expression of YAP stops in the cap stage. And the prolonged activation of PEK contributes to the formation of the ectopic cusp.^413^ Besides, in orthodontic tooth movement, there are diverse mechanisms for YAP/TAZ involved in periodontal tissue remodeling.^414^ Currently, although the roles of YAP/TAZ in dental and craniofacial development and remodeling have attracted wide interests, the role of YAP/TAZ is not well understood and some mechanisms are based on speculation, so further research is needed to explore their functions in‐depth.

**FIGURE 7:**
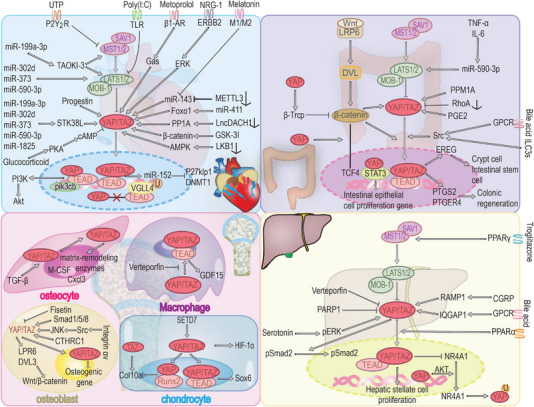
Different mechanisms of YAP/TAZ in organ regeneration. In heart, intestinal, liver, YAP/TAZ can be activated through Hippo‐dependent and Hippo‐independent way and then promotes the pro‐proliferation genes expression to stimulate cell proliferation, which contributes to heart, intestinal, and liver regeneration. And in bone, YAP/TAZ has an effect on cartilage regeneration and bone remodeling through regulating osteocyte, osteoblast, osteoclast, and chondrocyte.

In conclusion, YAP/TAZ has the ability to regulate cell proliferation to promote various organs regeneration through diverse signaling pathways (Figure 7). Even so, it's necessary to control the limit of cell proliferation to avoid uncontrolled, unlimited, and accelerated multiplication, which may result in tumors. Therefore, researching the function of YAP/TAZ with a dialectical view is needful.

## 8 THE CLINICAL EXPLORATIONS TARGETING YAP/TAZ

In fact, there are few specific inhibitors for YAP/TAZ at present, and most of them are in the stage of cell experiments or animal experiments. YAP/TAZ inhibitors that have been listed or entered clinical trials are very limited. VP is a listed YAP inhibitor approved by United States Food and Drug Administration (US FDA), which is currently mostly used in macular lesions. The study found that the target gene of YAP/TAZ was lowered after the use of VP. Further exploration of the mechanism found that VP can hinder the interaction between YAP/TAZ and TEAD and promote the transfer of YAP/TAZ from the nucleus to the cytoplasm, thus making YAP/TAZ unable to exert its effect.^415^ Although VP is listed as an eye disease drug, due to the involvement of YAP/TAZ in the development of many diseases, VP is also considered to play an important role in cancer, fibrosis, and other diseases, but more clinical trials are needed to prove the pharmacological effect of VP in other diseases.^416^ At present, most of the drugs in clinical trials of YAP/TAZ are targeted to the combination of YAP/TAZ and TEAD and are both in stage I or II of solid tumor clinical trials (Table 2). It is worth noting that some YAP inhibitors have been proven to be effective in cell and animal experiments and have been sold for researchers to study. Super‐TDU has been proven to be a competitive inhibitor of YAP, which can compete with YAP and combine with TEAD. The study found that Super‐TDU can not only significantly selectively inhibit the proliferation of human primary gastric cancer cells, but also reduce the number of tumors in the mouse model of Helicobacter pylori gastric cancer.^417^ In addition, CA3 has also been found to have significantly better YAP inhibition than VP and other small molecule inhibitors. CA3 has excellent selectivity and inhibition for cells with high YAP1 expression. CA3 can reduce the expression of YAP1 and weaken the transcriptional activity of YAP1 in a dose‐dependent way, thus significantly hindering the proliferation and differentiation of cancer stem cells and reducing the volume of tumors in the transplanted tumor model of esophageal adenocarcinoma mice.^418^ However, although Super‐TDU and CA3 have been shown to have good antitumor effects in mouse models, the two drugs lack corresponding clinical trials. Whether they are safe enough to be applied to the human body requires further clinical research to prove it.

**TABLE 2:** The drugs in clinic trial targeting YAP/TAZ.

NCT number	Drug name	Condition or disease	Clinical phase
NCT05228015	IK‐930	Solid tumors, adult solid tumor, mesothelioma, epithelioid hemangioendothelioma (EHE), NF2‐deficient mesothelioma, other NF2‐deficient solid tumors and solid tumors with YAP1/TAZ fusion genes, NF2 deficiency, YAP1 or TAZ gene fusions	Phase 1
NCT04857372	IAG933	Mesothelioma	Phase 1
NCT02416427	Atorvastatin	Breast neoplasms	Phase 2
NCT04659096	ION537	Advanced solid tumors	Phase 1
NCT04665206	VT3989	Solid tumor, adult mesothelioma	Phase 1

Data sources: ClinicalTrials.gov.

## 9 CONCLUSION AND OUTLOOK

As the graphical abstract shows, YAP/TAZ lies at the center of a regulatory network as a core component. On one hand, YAP/TAZ can serve as a downstream target regulated by various signaling pathways. On the other hand, YAP/TAZ also plays a key role as an upstream signal in diverse fields. In this review, we summarized the different signaling pathways regulating YAP/TAZ and the epigenetic regulation and posttranslational modification of YAP/TAZ. Under these controls, YAP/TAZ can shuttle between the nucleus and the cytoplasm. In the cytoplasm, YAP/TAZ is degraded, and in the nucleus, YAP/TAZ can bind to the TEAD to regulate the expression of its downstream target gene, thus affecting cell growth, proliferation and differentiation. In addition, the different effects of YAP/TAZ mediated are also summarized. Overactivation of YAP/TAZ may cause a variety of substance metabolic disorders and then cause metabolic syndrome. It can also activate the inflammatory genes of endothelial cells and macrophages, leading to the occurrence of atherosclerosis. More importantly, YAP/TAZ is involved in the occurrence of multiple organ fibrosis. YAP/TAZ can activate the transformation of fibroblasts into muscle fibroblasts, thus promoting the deposition of collagen. In fact, YAP/TAZ has a dual effect on the regulation of cell proliferation. On the one hand, for aging and damaged organs, the promotion of YAP/TAZ on cell proliferation makes organ regeneration possible. On the other hand, the excessive uncontrolled cell proliferation may also lead to the development of tumors. At the same time, YAP/TAZ will also enhance the resistance of tumors to anticancer drugs, greatly reducing the effect of chemotherapy. It is worth noting that a number of drugs have been shown to inhibit the activity of YAP/TAZ, which may play a role in the treatment of atherosclerosis and organ fibrosis (Table 3). In addition, as a YAP/TAZ‐specific inhibitor, it has been approved by the US FDA and has been tried to be applied in cancer and fibrosis. We also summarized the YAP/TAZ inhibitors currently in clinical trials. In short, we hope to improve new ideas for disease treatment targeting YAP/TAZ through this article.

**TABLE 3:** Summaries of drugs targeting YAP/TAZ in atherosclerosis and fibrosis.

Disease	Drug	Target	References
Atherosclerosis	Methotrexate	AMPK/YAP	212
	Sal‐B	YAP/TAZ/JNK	213
	Naringenin	YAP/TAZ/JNK	214
Myocardial fibrosis	Lovastatin	HMG‐CoA/YAP	222
	A reduced graphene oxide (rGO)/silk fibroin‐modified nanofibrous cardiac patch	YAP/TAZ‐TGF‐β1/Smads	223
Lung fibrosis	Simvastatin	Mevalonate pathway	240
	Melatonin	GPCR/Hippo pathway	243
	ICA	Hippo pathway	245
	Carthamin	Hippo pathway	246
Liver fibrosis	YQHX	YAP/TAZ	267
	DHI	YAP/TEAD	268
	Resveratrol	Hippo pathway	271
	Liquiritigenin	LATS1	272
	Morin	MST	273
	TKF	YAP/Notch	274
	MgIG	LATS	275
	MitoQ	JNK/YAP	276
	Pantoprazole	YAP	277
	Chlorella extract	LATS, Foxo1/p‐MAPK	279
Renal fibrosis	Verteporfin	YAP	279
	Polydatin	YAP	310

In fact, YAP/TAZ has been widely studied in recent years, and its mechanism of action and upstream and downstream regulation have become clearer. Despite extensive research on YAP/TAZ, there is only one YAP/TAZ‐specific inhibitor that can be applied to the human body, that is, VP. However, VP is primarily used for macular diseases rather than for the treatment of organ fibrosis or cancer. Most of the current research on YAP/TAZ inhibitors focuses on cell or animal experiments, with only a few YAP/TAZ inhibitors entering clinical trials none reaching clinical phase III drug studies. Many of the inhibitors applied to YAP/TAZ are “repurposed drugs,” such as statins. Therefore, there is an urgent need for the development of specific inhibitors targeting YAP/TAZ. Future studies can employ high‐throughput drug screening to identify more YAP/TAZ inhibitors and investigate their safety, toxicity, efficacy, and potential applications in humans. Furthermore, based on the existing pharmacophore of YAP/TAZ‐specific inhibitors, more selective and effective drugs can be designed. YAP/TAZ has been found to play a role in cancer and fibrosis in many organs. In fact, studies have shown that liver fibrosis and pulmonary fibrosis can gradually progress to liver cancer and lung cancer. Therefore, further research is needed to explore whether the expression level of YAP/TAZ has changed during the transformation of fibrosis into cancer and whether YAP/TAZ mediated the malignant transformation of fibrosis. In addition, due to the high expression of YAP/TAZ in many diseases, it can be used as a biomarker for disease diagnosis. The YAP/TAZ simple test kit deserves to be designed and developed for early diagnosis of disease.

In conclusion, YAP/TAZ plays an important regulatory role in diverse diseases and is regulated by various signaling pathways. Targeting YAP/TAZ and its signaling pathways may offer new avenues for disease treatment and drug discovery. Exploring the upstream and downstream signaling of YAP/TAZ can further broaden our understanding of disease mechanisms and potential therapeutic interventions.

## AUTHOR CONTRIBUTIONS

All authors contributed to the research idea and design of this study. Yuzi Wei conceived and wrote the manuscript. Yilin Chen and Ruiying Han participated in the design of the figures. Victoria Lee Zhi Hui and Xianglong Han revised the manuscript. Yongwen Guo conceived the study and revised the manuscript finally. All authors have read and approved the final manuscript.

## CONFLICT OF INTEREST STATEMENT

There is no conflict of interest for all the authors.

## ETHICS STATEMENT

Not applicable.

## Data Availability

Not applicable.
